# Functional Characterization of *Aspergillus nidulans ypkA,* a Homologue of the Mammalian Kinase SGK

**DOI:** 10.1371/journal.pone.0057630

**Published:** 2013-03-05

**Authors:** Ana Cristina Colabardini, Neil Andrew Brown, Marcela Savoldi, Maria Helena S. Goldman, Gustavo Henrique Goldman

**Affiliations:** 1 Laboratório Nacional de Ciência e Tecnologia do Bioetanol – CTBE, São Paulo, Brazil; 2 Faculdade de Ciências Farmacêuticas de Ribeirão Preto, Universidade de São Paulo, São Paulo, Brazil; 3 Faculdade de Filosofia, Ciências e Letras de Ribeirão Preto, Universidade de São Paulo, São Paulo, Brazil; University of Wisconsin - Madison, United States of America

## Abstract

The serum- and glucocorticoid-regulated protein kinase (SGK) is an AGC kinase involved in signal cascades regulated by glucocorticoid hormones and serum in mammals. The *Saccharomyces cerevisiae ypk1* and *ypk2* genes were identified as SGK homologues and Ypk1 was shown to regulate the balance of sphingolipids between the inner and outer plasma membrane. This investigation characterized the *Aspergillus nidulans YPK1* homologue, YpkA, representing the first filamentous fungal *YPK1* homologue. Two conditional mutant strains were constructed by replacing the endogenous *ypk1* promoter with two different regulatable promoters, *alcA* (from the alcohol dehydrogenase gene) and *niiA* (from the nitrate reductase gene). Both constructs confirmed that *ypkA* was an essential gene in *A. nidulans*. Repression of *ypkA* caused decreased radial growth, a delay in conidial germination, deficient polar axis establishment, intense branching during late stages of growth, a lack of asexual spores, and a terminal phenotype. Membrane lipid polarization, endocytosis, eisosomes and vacuolar distribution were also affected by *ypkA* repression, suggesting that YpkA plays a role in hyphal morphogenesis via coordinating the delivery of cell membrane and wall constituents to the hyphal apex. The *A. nidulans* Pkh1 homologue *pkhA* was also shown to be an essential gene, and preliminary genetic analysis suggested that the ypkA gene is not directly downstream of *pkhA* or epistatic to *pkhA*, rather, *ypkA* and *pkhA* are genetically independent or in parallel. *BarA* is a homologue of the yeast *Lag1* acyl-CoA-dependent ceramide synthase, which catalyzes the condensation of phytosphingosine with a fatty acyl-CoA to form phytoceramide. When *barA* was absent, *ypkA* repression was lethal to the cell. Therefore, there appears to be a genetic interaction between *ypkA*, *barA*, and the sphingolipid synthesis. Transcriptional profiling of *ypkA* overexpression and down-regulation revealed several putative YpkA targets associated with the observed phenotypes.

## Introduction

Protein kinases are key regulators of cellular function that act via phosphorylating target proteins thus directing their activity, location and function. Serum- and glucocorticoid-regulated protein kinases (SGK) are members of the AGC kinase subfamily (PKA, PKC, PKC) and are involved in signal cascades which are regulated in mammals by serum and glucocorticoid hormones [1]. Additionally, the mammalian SGK is activated in response to the follicle-stimulating hormone that is produced during brain damage [2] and the transfection of mammary epithelial cells with the p53 transcription factor [3]. The threonine residue (Thr^256^), located on the SGK activation loop of the catalytic domain, is phosphorylated by the 3-phosphoinositide-dependent protein kinase-1 (PDK1), which serves as a central integrator of signaling cascades. Additional PDK1 targets include the p70 S6 kinase, PKC isoforms, PKB and SGK [4]. Two *S. cerevisiae* genes, *ypk1* and *ypk2*, were identified as SGK homologues. The *ypk1* was shown to confer resistance to ISP-1, an inhibitor of sphingolipid synthesis [5]. Complementation of the *Δypk1 S. cerevisiae* strain with the mammalian SGK restored viability. The *S. cerevisiae pkh1* protein kinase possesses a catalytic domain that resembles the mammalian PDK1 catalytic domain. In *S. cerevisiae*, Pkh1 is essential. Complementation of the *Δpkh1* with PDK1 restored viability, while Ypk1 was also demonstrated to be phosphorylated by Pkh1 on the Thr^504^ residue, indicating that PDK1 and *pkh1* are functional homologues [6].

Sphingolipids are essential components of eukaryotic cells providing membrane structure, acting as secondary messengers and interacting with sterols to form lipid-rafts [7], [8]. In eukaryotic cells, sphingolipids are also involved in important cellular processes such as endocytosis [9], [10], intracellular trafficking of proteins and lipids [11], [12], cell cycle control [13], [14], and the heat shock response [15], [16]. In *S. cerevisiae* Ypk1 regulates two flipase protein kinases, Fpk1 and Fpk2, responsible for maintaining the balance between sphingolipids in the inner and outer plasma membrane by activating flipases proteins, which maintain layer asymmetry through the expulsion of amino phospholipids from the outer layer. Ypk1 negatively regulates Fpk1, thus the *ypk1* null mutant possesses defects that result from flipases hyper-activity which are deleterious to cell viability [17]. In mammals, two well characterized secondary messengers, which are derived from sphingolipids, sphingosine 1-phosphate and ceramides, are both involved in growth and apoptosis signaling [18], [19]. In *S. cerevisiae*, phytosphingosine activates Pkh1 which in turn activates Ypk1 [20]. Ypk1 is responsible for the inactivation of two endoplasmic reticulum membrane proteins, Orm1 and Orm2, which inhibit the responsible for the first catalytic step of the sphingolipid biosynthesis [21].

The integral highly conserved serine/threonine protein kinase, target of rapamycin (TOR), forms two complexes (TORC1 and TORC2) that regulate cell growth and metabolism in response to the environment. AGC kinases are activated by phosphorylation of the activation loop, turn motif and hydrophobic motif [22]. Hydrophobic motif phosphorylation on the Ypk1 (Thr 662) is mediated by TORC2, which regulates cytoskeleton organization, [23] and this phosphorylation site is required for the resistance to myriocin, an inhibitor of sphingolipid synthesis. In response to sphingolipids depletion, the *S. cerevisiae ypk1^T662A^* mutant has low Orm phosphorylation *in vivo*, as well as, low activation *in vitro*. Thus Ypk1 is both a sensor and an effector of sphingolipids levels, with sphingolipid reduction, at least in part stimulating Ypk1 via TORC2 mediated phosphorylation [21]. Besides the interaction with TORC2, Ypk1 also interacts with TORC1. The TORC1 complex positively regulates translation initiation, biogenesis of ribosomes, and the uptake of amino acids through the sensing of nutrient availability. Protein translation is rapidly interrupted in response to a lack of nitrogen through the autophagic proteolysis of Ypk1 [24]. TORC1 and Ypk1 are therefore differentially controlled by the lack of nitrogen, but share the same downstream targets, such as the translation initiation factor eIF4G [25].

The highly polarized nature of the fungal cells is a hallmark of their morphology as they grow through the insertion of a new membrane into the cell wall surface. The tubular cell shape is due to the fact that growth is confined to hyphae apical hub [26]. For this purpose, vesicles loaded with components required for the cell wall expansion are transported to active sites of growth over a network of polarized microtubes [27]. Therefore, polarized growth requires proteins involved in cytoskeleton functions and secretory endocytic machinery [28]. The plasma membrane consists of different sub-domains defined by its distribution of sphingolipids and sterols. These sphingolipids can be grouped into sub-domains, rather than being distributed homogeneously throughout the glyceroglycolipid membrane [29]. Sterols are four-ring structures that possess an aliphatic tail, which may “pack” sphingolipids together [30]. These “packs”, referred to as lipid-rafts, play an important role in protein localization and signal transduction. Lipid-rafts serve as mounting and organizing centers for signaling molecules and are also very important for polar organization of the cell [31].

Lipids have also been implicated as performing a role in membrane trafficking [32]. In mammalian cells, sphingosine induces the phosphorylation of certain protein kinases *in vitro* [33]. In *S. cerevisiae*, sphingolipids induce Pkh1 auto-phosphorylation, which in turn activates several other proteins kinases, including Ypk1 [6]. Mutations that block the early stages of endocytosis show a reduction in the assimilation of the fatty acids, indicating that Ypk1 affects the uptake of fatty acids by regulating endocytosis [34]. In addition to signaling events and vesicular transport, the control of cell growth involves the synthesis of a new cell wall skeleton and the reorganization of actin. The actin cytoskeleton directs the delivery of components to the hyphal apex and it is essential for maintaining polarized growth. In fungi, the cell wall determines the shape of, and confers rigidity to, the cell. Furthermore, the cell wall is a dynamic structure that functions as a sensor of turgor and is continuously remodeling in response to environmental stimuli and growth [35]. The *S. cerevisiae* protein kinase C (Pkc1) activates the cell wall maintenance MAP kinase signaling cascade in response to osmotic stress and restrictive temperatures, mediating the signal from TORC2, to regulate the actin cytoskeleton [36]. Pkh2 and possibly Pkh1 phosphorylate and activate Pkc1 [37]. In addition, *ypk* mutants demonstrate a random distribution of the actin cytoskeleton and are deficient in the activation of MAP cascade [38].

This study presents the first *YPK1* homologue, *ypkA*, to be characterized in a filamentous fungus. In *A. nidulans*, *ypkA* was shown to be an essential gene that is involved in polar axis establishment and germling growth. Membrane lipid polarization was deficient when the *ypkA* conditional mutant was grown in repressing conditions, suggesting that YpkA plays an important role in hyphal morphogenesis that also affects endocytosis and vacuolar distribution in *A. nidulans*.

## Results

### The *A. nidulans* ypkA Gene is Essential

**Figure 1:**
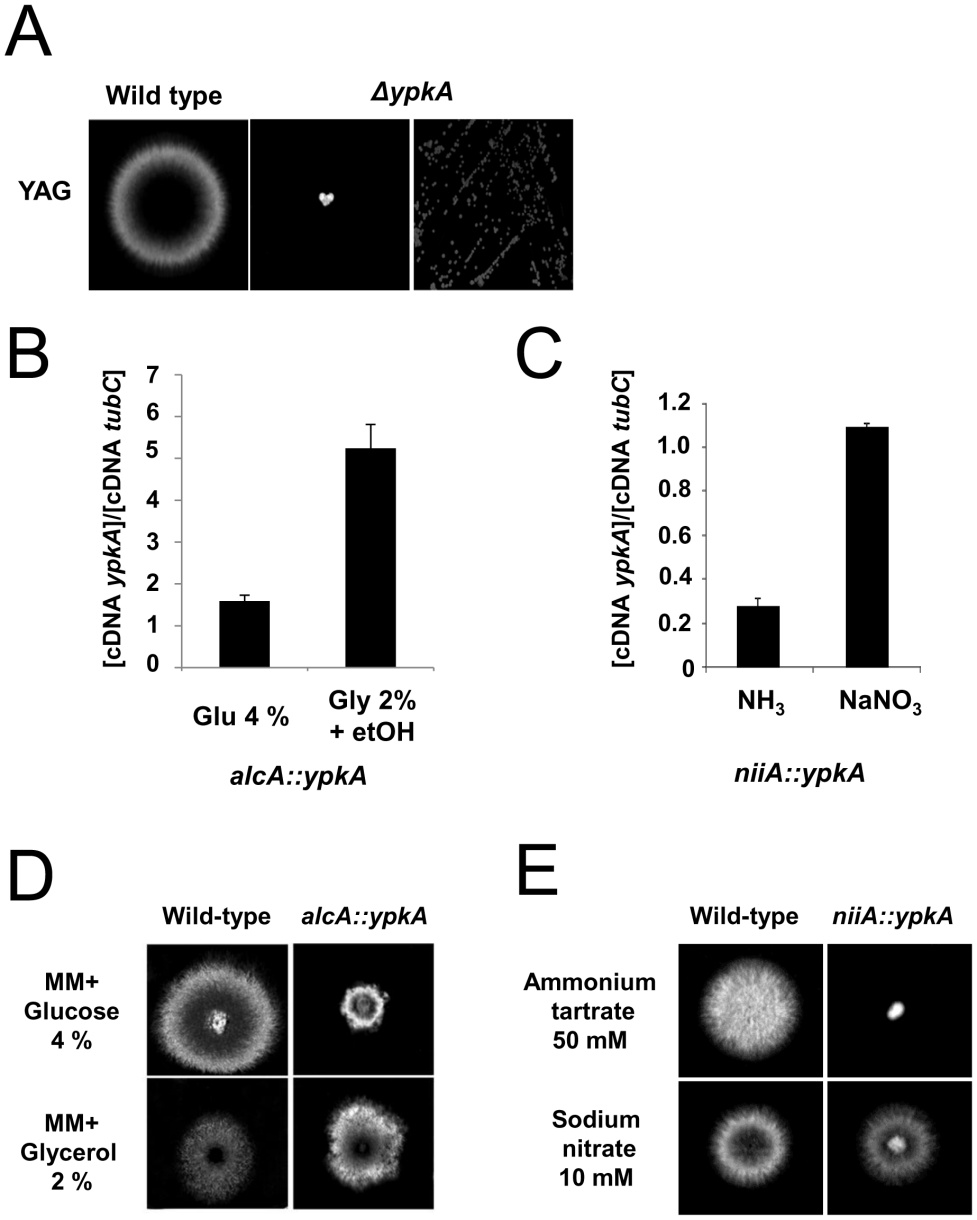
The *ypkA* gene is to essential *A. nidulans*. (A) Wild-type and primary *ΔypkA* transformant were grown (left and center panel) or streaked (right panel) on YAG medium for 96 hours at 37°C. (B) The *alcA::ypkA* strain was grown for 6 hours in MM+4% glucose or MM+2% glycerol +threonine 100 mM (C) The *niiA::ypkA* strain was grown for 6 hours in MM+sodium nitrate 10 mM or MM+ammonium tartrate 50 mM. The relative quantitation of *ypkA* and tubulin gene expression was determined by a standard curve (i.e., C_T_ –values plotted against a logarithm of the DNA copy number). The presented results are the means (± standard deviation) of four biological replicates. The growth phenotypes of the *alcA::ypkA* (D) and *niiA::ypkA* (E) mutant strains. The *A. nidulans* wild-type, *alcA::ypkA*, and *niiA::ypkA* mutant strains were grown for 72 hours at 37°C either on MM+4% glucose or MM+2% glycerol and MM+sodium nitrate 10 mM or MM+ammonium tartrate 50 mM.

A BLASTp search of the *A. nidulans* genome database (http://www.aspgd.org) using the *S. cerevisiae* Ypk1p and Ypk2p as queries revealed a single open reading frame (ORF) with significant similarity. The potential homologue, AN5973 (here named *ypkA*), is predicted to be a 640-amino acid protein kinase with high identity to Ypk1p (2e−135; 47.5% identity and 62.8% similarity) and Ypk2 (1.1e−134; 47.8% identity; 62.1% similarity) mainly within the C-terminal region. YpkA has two defined domains, a protein kinase domain (PS50011) from residues 293 to 550 and an AGC-kinase C-terminal domain (PS51285) from residues 551 to 622. An *in vivo S. cerevisiae* fusion-based approach (see Materials and Methods) was used in an attempt to generate an *A. nidulans ypkA* null mutant. Several primary transformants were isolated. However, these transformants displayed a pronounced phenotypic defect on MM, i.e. very small non-sporulating colonies (Figure 1A; middle and right panels). These colonies were unable to grow or produce a significant amount of mycelium in liquid medium, suggesting a terminal phenotype for these strains. Heterokaryon rescue [39] confirmed that *ypkA* is an essential gene (data not shown). Subsequently, two conditional mutants for *ypkA* were constructed by replacing the endogenous *ypkA* promoter with the either the *alcA* or *niiA* promoters. The *alcA* promoter is repressed by glucose, derepressed by glycerol and induced to high levels by ethanol or L-threonine [40]. Transformants that accumulated approximately 3-fold higher *ypkA* mRNA when transferred to glycerol 2%+threonine than when transferred to glucose 4% were selected (Figure 1B). The second promoter from *niiA* (encoding a nitrite reductase) is induced by sodium nitrate and repressed by ammonium tartrate [41]. Transformants that showed an approximate four-fold decrease in *ypkA* mRNA accumulation when transferred to ammonium tartrate than when transferred to sodium nitrate were selected (Figure 1C).

Repression by growing the *alcA::ypkA* mutant strain in the presence of 4% glucose decreased colony diameter approximately four-fold (Figure 1D). In contrast, both wild-type and *alcA::ypkA* strains had the same radial diameter when grown in 2% glycerol (Figure 1D). Overexpression of *ypkA* did not cause any detectable phenotypic change (data not shown). Repression by growing the *niiA::ypkA* mutant strain in the presence of ammonium tartrate caused a dramatic ten-fold decrease in the colony diameter (Figure 1E). These results strongly indicate *ypkA* is an essential *A. nidulans* gene. The construction of these two strains, with different levels of YpkA expression, was desired to enable different experimental approaches that would assist in the investigation of YpkA function. For instance, the *niiA* promoter allows very little leakage during repression and can be used to study the impact of YpkA loss of function while, the *alcA* promoter allows the study of the *ypkA* overexpression.

**Figure 2:**
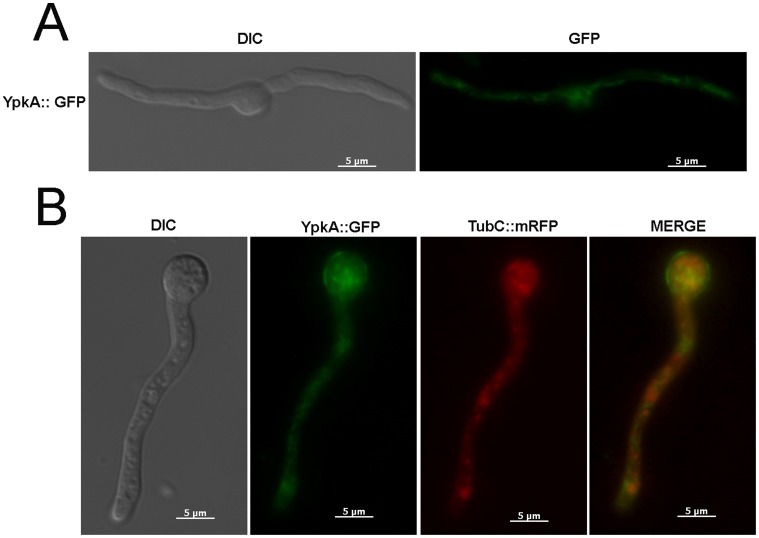
YpkA::GFP partially co-localizes with TubC::mRFP. Conidia were grown for 16 hours at 30°C in YG medium, YpkA::GFP (A) and YpkA::GFP TubC::mRFP (B). Bars, 5 µm.

To evaluate the sub-cellular localisation of YpkA, a YpkA::GFP strain was constructed. The YpkA::GFP strain behaved exactly the same as the wild-type strain (data not shown). When the YpkA::GFP strain was grown in YG for 16 hours at 30^o^C, disperse fluorescence was observed in the cytoplasm that appeared similar to the distribution of tubulin (Figure 2A). To test this hypothesis, a double YpkA::GFP TubC::mRFP strain was constructed by crossing the corresponding parental strains. Partial co-localization of the two proteins was observed (Figure 2B).

### *A. nidulans* YpkA is Involved in the Polar Growth and Endocytosis

**Figure 3:**
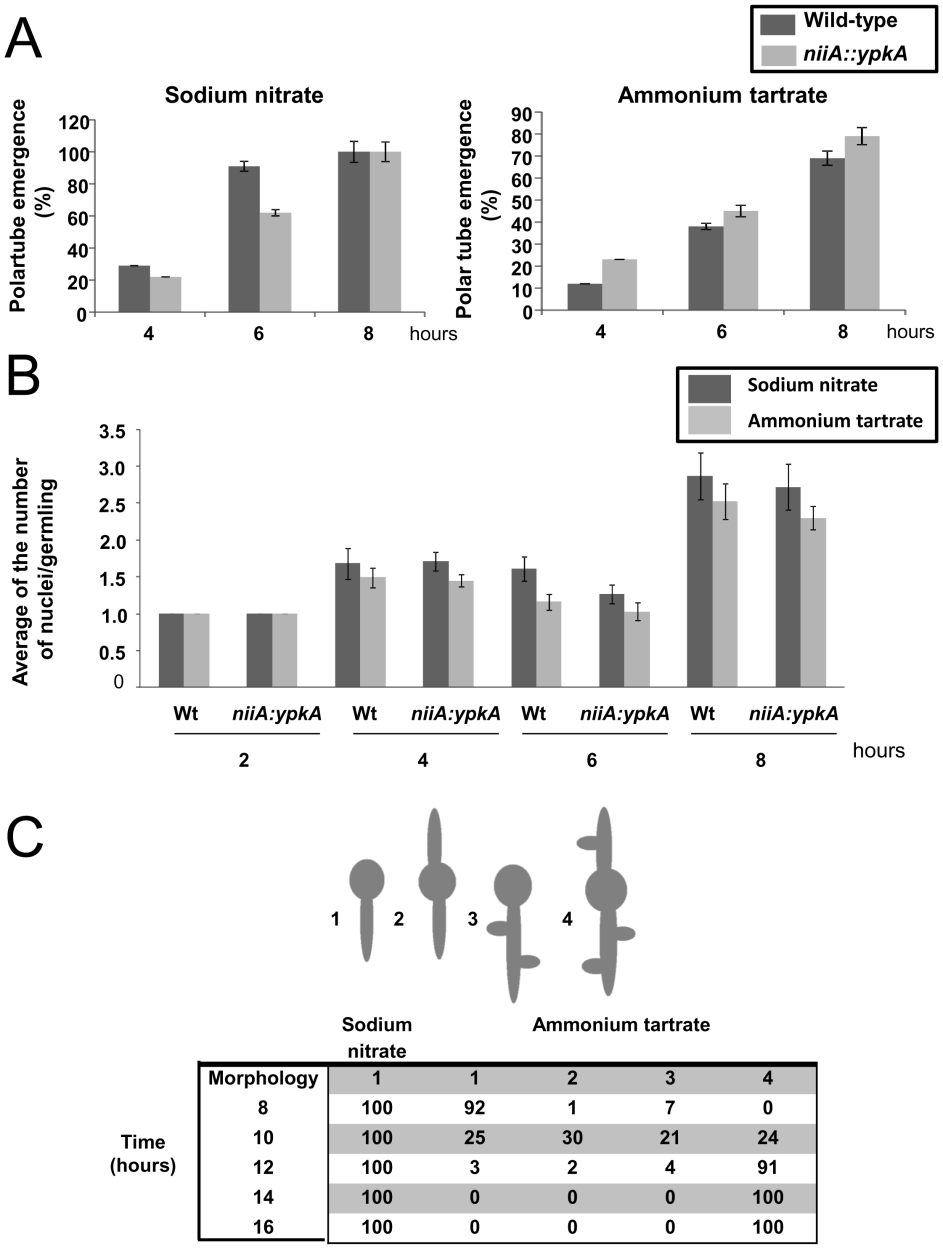
*A. nidulans* YpkA is involved in polarized growth. The percentage of wild-type and *niiA::ypkA* mutant germlings that exhibited polar growth (defined here as the emergence of the germ tube) (A) and the number of nuclear per germling (B). Conidia were grown for 2 to 8 hours at 37°C. Averages (± standard deviation) represent 100 germlings from three independent experiments (Wt = Wild-type). (C) The germination pattern of the wild-type and *niiA::ypkA* conidiospores. Conidia were allowed to germinate on MM media for 8 to 16 hours. Conidia possessing germ tubes were classified as displaying (left to right) unipolar (1), bipolar (2), unipolar plus lateral branches (3) or bipolar plus lateral branches (4).

**Figure 4:**
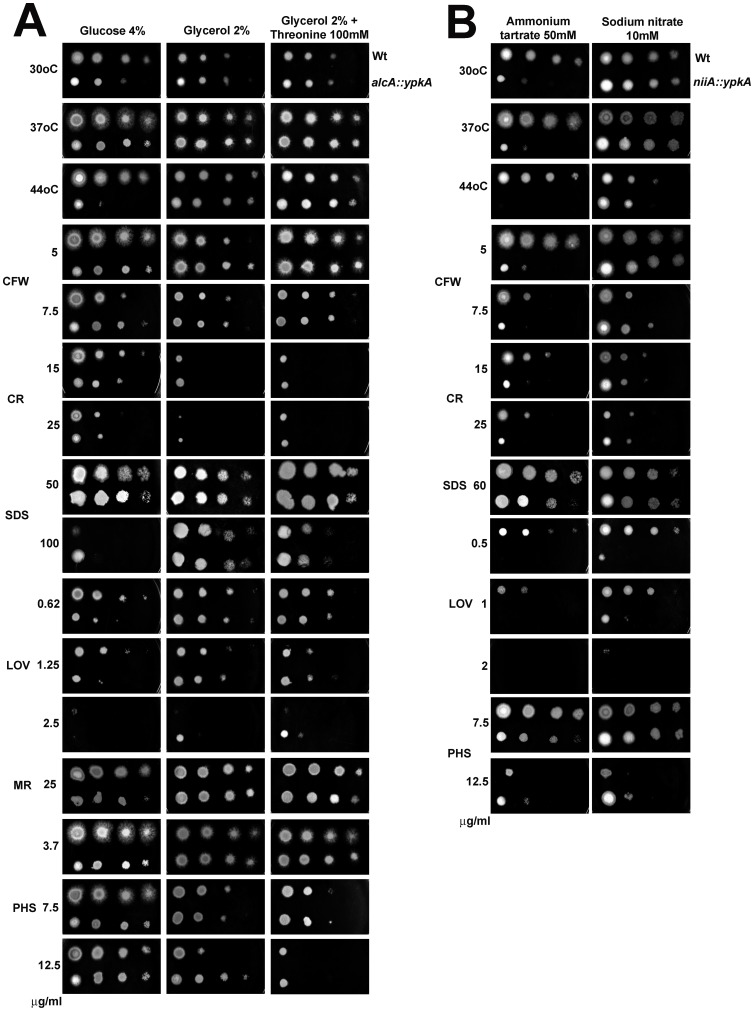
Evaluation of the effect of YpkA depletion and overexpression on the growth of *A. nidulans* under different experimental conditions. Five µl of a ten-fold dilution series starting at a concentration of 2×10^7^ for the wild-type (A-D), *alcA::ypkA* (A-B), and *niiA::ypkA* (C–D) strains were spotted on different growth media and grown for 72 hours at 37°C, except for experiments where the temperature was evaluated (A and C). CFW = calcofluor white; CR = congo red; MR = myriocin; PHS = phytosphingosine; and SDS = sodium dodecyl sulphate.

**Figure 5.The:**
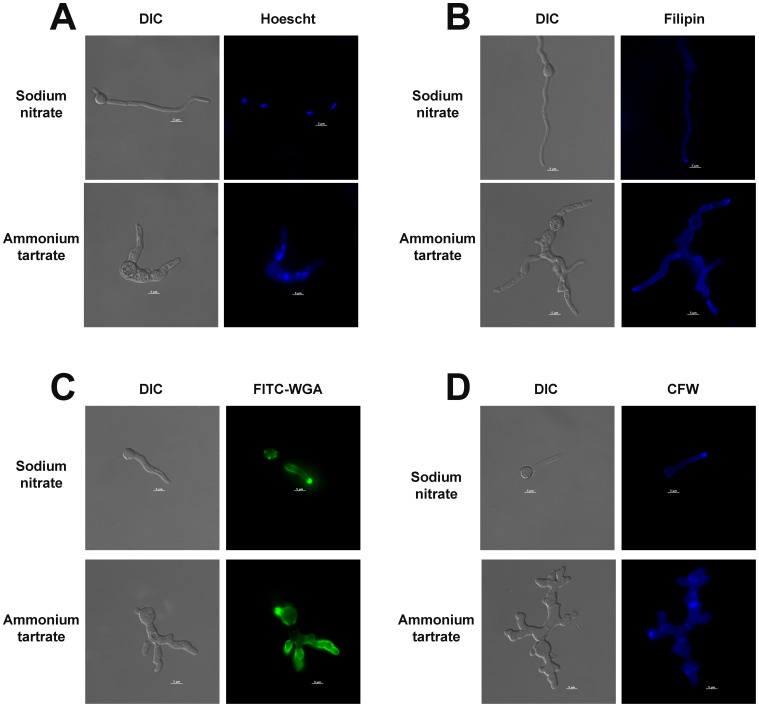
polarized delivery of membrane lipids and cell wall deposition was not confined to the hyphal apex in the *niiA::ypkA* mutant upon repression. In all experiments, germlings were grown for 16 hours at 37°C on inducing (sodium nitrate) and repressing conditions (ammonium tartrate). Stains utilized: (A) Hoescht, (B) Filipin, (C) FITC-conjugated wheat germ, and (D) CFW. Bars, 5 and 10 µm.

The morphogenetic program associated with the germination of *A. nidulans* conidiospores includes; (i) a brief period of isotropic spore swelling, (ii) the establishment of a stable polarity axis, and (iii) the emergence of a polarized germ tube [42]. This program leads to the formation of a multinucleate hyphal cell that grows by apical extension. We assess if YpkA affects polar growth, germ-tube emergence and the number of nuclei within the wild-type and *niiA::ypkA* hyphae grown either in the presence of sodium nitrate or ammonium tartrate (Figures 3A–B). A delay in germ-tube emergence was observed when the wild-type strain was grown in media containing ammonium tartrate (Figure 3A). No significant difference in germ-tube emergence or the number of nuclei was observed between the wild-type and the *niiA::ypkA* mutant strains in the first eight hours of germination (Figures 3A–B and 4A). However, the nuclei of the *niiA::ypkA* mutant grown under repressing conditions appeared fragmented (Figure 5A). The overexpression of YpkA did not affect germ-tube emergence or the number of nuclei in the first eight hours of germination (data not shown). When the *niiA::ypkA* strain was grown for longer periods (10 to 16 hours) in the presence of sodium nitrate, 100% of germ-tubes showed a single polar axis (Figure 3C). However, we observed multiple polar axes when *niiA::ypkA* strain was grown in the presence of ammonium tartrate (Figure 3C). These results suggest that YpkA might play a role in recruiting the morphogenetic machinery, including for instance several components of a multiprotein complex termed the polarisome [26] to the site of polarization.

Subsequently, the two constructed strains were used to investigate the effects of *ypkA* repression and overexpression under different experimental conditions (Figure 4). Under repressing conditions plus high temperature (44°C) or the presence of lovastatin, growth of the *alcA::ypkA* strain was reduced to a greater extent than the wild-type strain (Figures 4A and B). However, under repressing conditions the *alcA::ypkA* strain was more resistant to SDS (Sodium Dodecyl Sulfate) and Calcofluor white (CFW) than the wild-type strain (Figure 4A). Surprisingly, under either repressing or overexpressing conditions, growth of the *alcA::ypkA* strain was not affected by myriocin and phytosphingosine (Figure 4B). Overexpression of *ypkA* slightly increased the resistance to lovastatin (Figure 4B). Accordingly, under repressing conditions, growth of the *niiA::ypkA* strain was also most highly affected by higher temperatures (44°C) and lovastatin, while being more resistant to SDS and CFW (Figures 4C and D). Again, under repressing conditions, the *niiA::ypkA* strain was not affected by phytosphingosine (Figure 4B).

Germlings of the wild-type and *niiA::ypkA* mutant strains were stained with filipin, a fluorescent polyene antibiotic that binds sterols [43], to determine whether membrane lipids were being delivered to the hyphal apex during polar growth (Figure 5B). Intense filipin staining was observed in the hyphal apex of the wild-type strain when grown either in the presence of sodium nitrate or ammonium tartrate (Figure 5B, upper panel). Filipin staining was localized to the hyphal apex of the *niiA::ypkA* mutant under inducing conditions, while staining was uniformly dispersed throughout the membrane under repressing conditions (Figure 5B, lower panel). In *A. nidulans*, the FITC-conjugated lectin WGA can be used to detect sites of cell wall deposition [26]. The confinement of FITC-WGA staining to the hyphal apex of the *niiA::ypkA* mutant, as observed under inducing conditions (Figure 5C, upper panel), was lost during *ypkA* repression (Figure 5C, lower panel). CFW staining demonstrated similar results, where CFW localization to the hyphal apex was lost, in the *niiA::ypkA* strain, under repressing conditions (Figure 5D). Additional septa were also noted in the *niiA::ypkA* germlings when grown under repressing conditions (Figure 5D). These observations suggest that the pool of vesicles carrying cell wall precursors were being inappropriately distributed along the hyphae of the mutant germlings under *ypkA* repression.

**Figure 6.YpkA:**
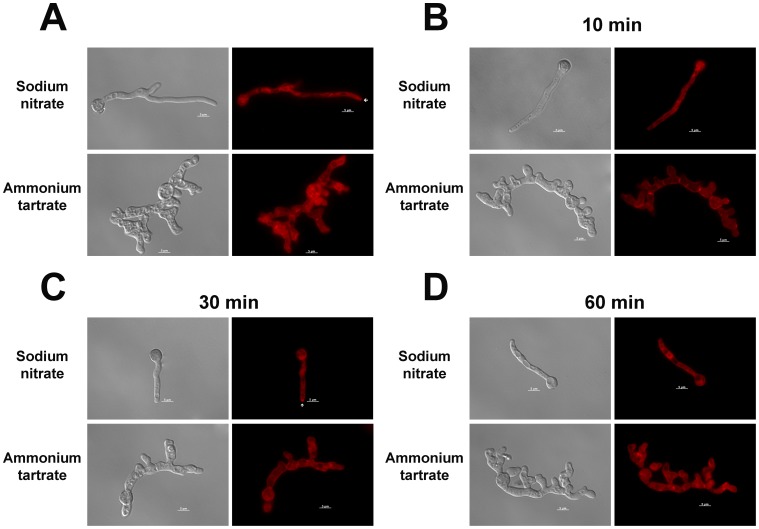
expression affects endocytosis and vacuolar distribution in *A. nidulans*. (A) Conidiospores were grown in glass-bottom dishes (Mattek Corporation, USA) with 2 ml of MM-G+supplements for 12 hours at 44°C and exposed to pre-warmed media containing 10 µM FM4-64 for 2 minutes. The coverslips were briefly rinsed in fresh media and incubated in MM-G without FM4-64 for 3 hours to allow for dye internalization and vesicle trafficking. The living cells were visualized at room temperature using a fluorescence microscope. (B, C, and D) show incubation of the FM4-64 for 10, 30, and 60 minutes. Arrows show the Spitzenkörper. Bars, 5 µm.

It has been demonstrated that *S. cerevisiae* Ypk1 acts downstream of the Pkh kinases to control endocytosis by phosphorylating components of the endocytic machinery [44]. Ypk1 and possibly the human Sgk1 kinase affect fatty-acid uptake and thus energy homeostasis through regulating endocytosis [34]. FM4-64 assays were performed to investigate intracellular trafficking, secretion, and vesicular transport. Under inducing conditions, FM4-64 staining revealed the Spitzenkörper at the hyphal apex and also structures that probably represent mature endosomes/vacuoles [45] in the wild-type and *niiA::ypkA* strains. In contrast, under repressing conditions the Spitzenkörper could not be visualized in the *niiA::ypkA* strain only and there was a significant decrease of the endosome/vacuole structures (Figure 6A). To verify the function of YpkA in endocytosis, live cells of the *niiA::ypkA* strain were stained with FM4-64 and the uptake of the dye tracked over time. Under inducing conditions, FM4-64 was visible on the plasma membrane and within the cell after 10 min, was taken up by cells and localized to endomembranes, which may be mature endosomes or vacuoles, after 30 min (Figure 6B–C). Under repressing conditions, FM4-64 uptake was delayed. After 10 min the dye remained on the plasma membrane and on structures that resembled septa (Figure 6B). Even after 60 min or more, staining of endomembrane remained diffuse (Figure 6D).

**Figure 7:**
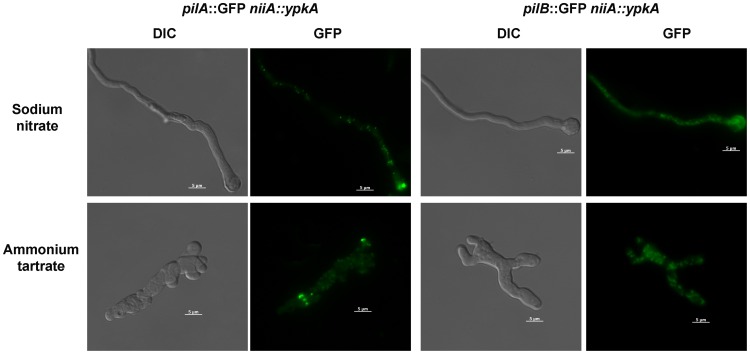
YpkA expression affects eisosomes distribution in *A. nidulans*. The *niiA::ypkA pilA::gfp* (A) and *niiA::ypkA pilB::gfp* (B) strains were grown for 16 hours at 37°C in MM+10 mM sodium nitrate or 50 mM ammonium tartrate. Bars, 5 µm.

Eisosomes are fungal subcortical organelles that play roles in endocytosis and the organization of the plasma membrane [32], [46]. They include two homologous proteins, Pil1 and Lsp1, which colocalize with the transmembrane protein Sur7 [32], [46]. In *S. cerevisiae* the Pkh1/2-Ypk1/2 signaling pathway regulates eisosome assembly and turnover [47]. Recently, the two homologues of Pil1/Lsp1, PilA and PilB, were identified in *A. nidulans* [48]. In *A. nidulans* wild-type mycelia, punctate structures composed of PilA are present, while PilB is diffused in the cytoplasm. The construction of *pilA::gfp* and *pilB::gfp* in the *niiA::ypkA* background enabled the evaluation of PilA and PilB localization upon *ypkA* induction and repression. When grown under inducing conditions, as previously observed in the wild-type strain, PilA localized to punctate structures in the cytoplasm, while PilB was diffused throughout the cytoplasm (Figure 7A). Upon *ypkA* repression, there was an increase in the punctate distribution of PilA and PilB throughout the cytoplasm (Figure 7B). These results suggest the depletion of YpkA may affect eisosome turnover, increasing the number of structures.

Taken together these results imply that *ypkA* performs an essential role in hyphal morphogenesis and filamentous growth, with the reduction in *ypkA* expression resulting in deficiencies in polarization related to endocytosis, vesicle transport and the polarized delivery of chitin/lipid to the hyphal apex.

### *A. nidulans* YpkA does not Interact with PkhA

**Figure 8:**
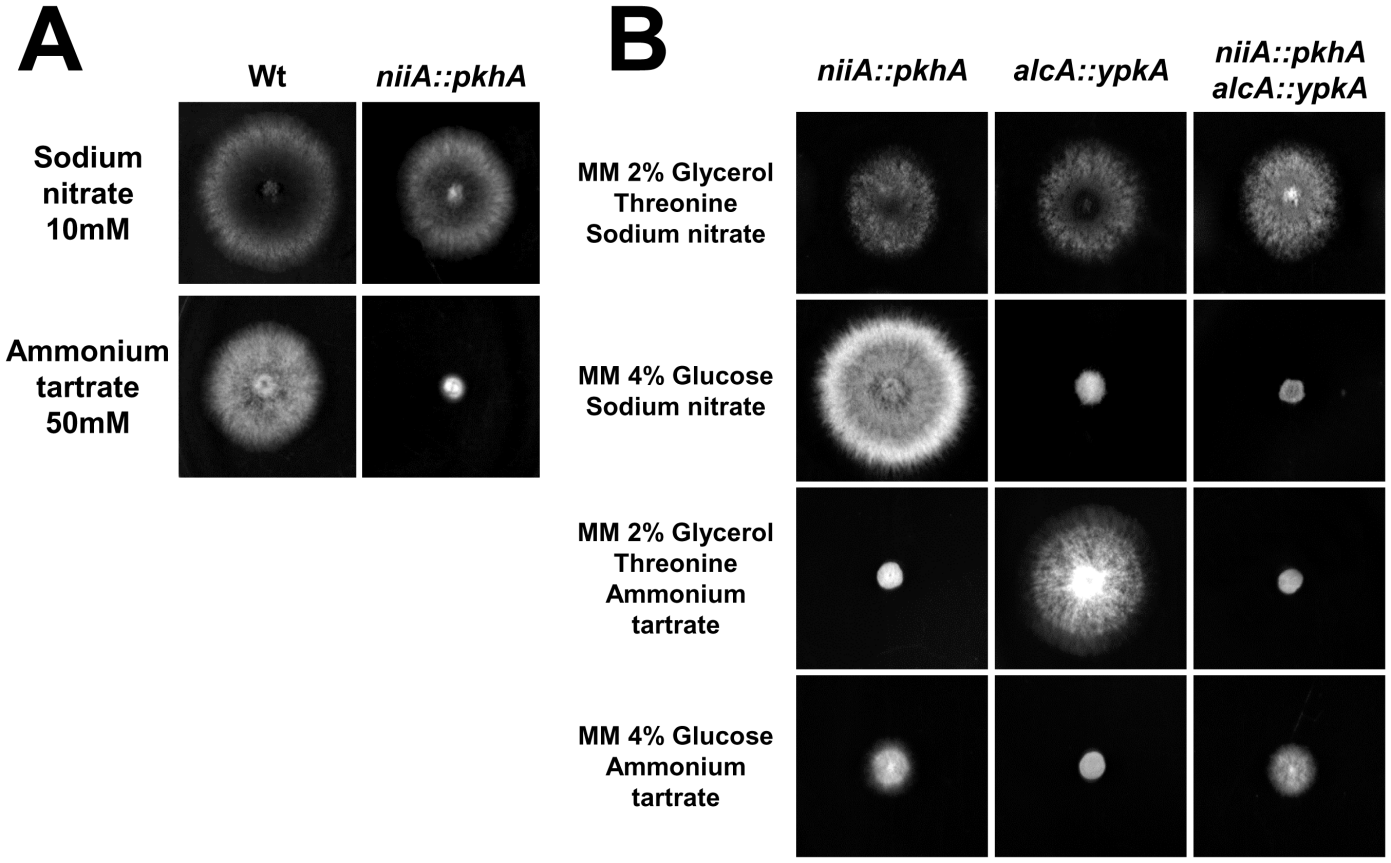
The *pkhA* gene is essential to *A. nidulans* and interacts with *ypkA*. (A) The wild-type and *niiA::pkhA* mutant strains were grown for 72 hours at 37°C on MM+sodium nitrate 10 mM or MM+ammonium tartrate 50 mM (Wt = Wild-type). (B) The *alcA::ypkA*, *niiA::pkhA*, and *alcA::ypkA niiA::pkhA* strains were grown for 72 hours at 37°C on different combinations of MM+glucose 2% or glycerol 2% plus threonine 100 mM plus sodium nitrate 10 mM or ammonium tartrate 50 mM (Wt = Wild-type).

In *S. cerevisiae*, Pkh1 activates Ypk1 [20]. Thus, as a first step to verify if an *A. nidulans* Pkh1 homologue interacts with the *A. nidulans* YpkA, a BLASTp search of the *A. nidulans* genome database (http://www.aspgd.org) using the *S. cerevisiae* Pkh1 as a query revealed a single ORF with significant similarity. The potential homologue, AN3110 (here named *pkhA*), is predicted to be an 813 amino acid with high identity to PkhA (8e−98; 449.8% identity and 69.4% similarity). PkhA has a well-defined protein kinase domain (PS50011). The generation of an *A. nidulans pkhA* null mutant, using an *in vivo S. cerevisiae* fusion-based approach (see Materials and Methods) was unable to generate any primary transformant (data not shown). Thus a conditional mutant for *pkhA* was constructed by replacing the endogenous *pkhA* promoter with the *niiA* promoter. When the *pkhA* was repressed, by growing the *niiA::pkhA* mutant strain in the presence of ammonium tartrate, there was a dramatic ten-fold decrease in the colony diameter (Figure 8A). These results strongly indicate that *pkhA* is also an essential *A. nidulans* gene. A *niiA::pkhA alcA::ypkA* double mutant was constructed. When the double mutant was grown on 4% glucose plus ammonium tartrate, representing *pkhA* and *ypkA* repressing conditions, radial growth was comparable to the radial growth of the *alcA::ypkA* mutant strain grown under the same conditions (Figure 8B). The *alcA::ypkA* strain showed a radial diameter similar to that of the double mutant in repressing conditions for *niiA* promoter and inducing conditions for *alcA* promoter (Figure 8B). The radial diameter of the double mutant was similar during growth on glucose plus sodium nitrate, representing *ypkA* repression (Figure 8B, second lane) and glucose plus ammonium tartrate, representing *pkhA* repression (Figure 8B). Taken together, these results suggested that the ypkA gene is not directly downstream of *pkhA* or epistatic to *pkhA*, rather, *ypkA* and *pkhA* are genetically independent or in parallel.

**Figure 9:**
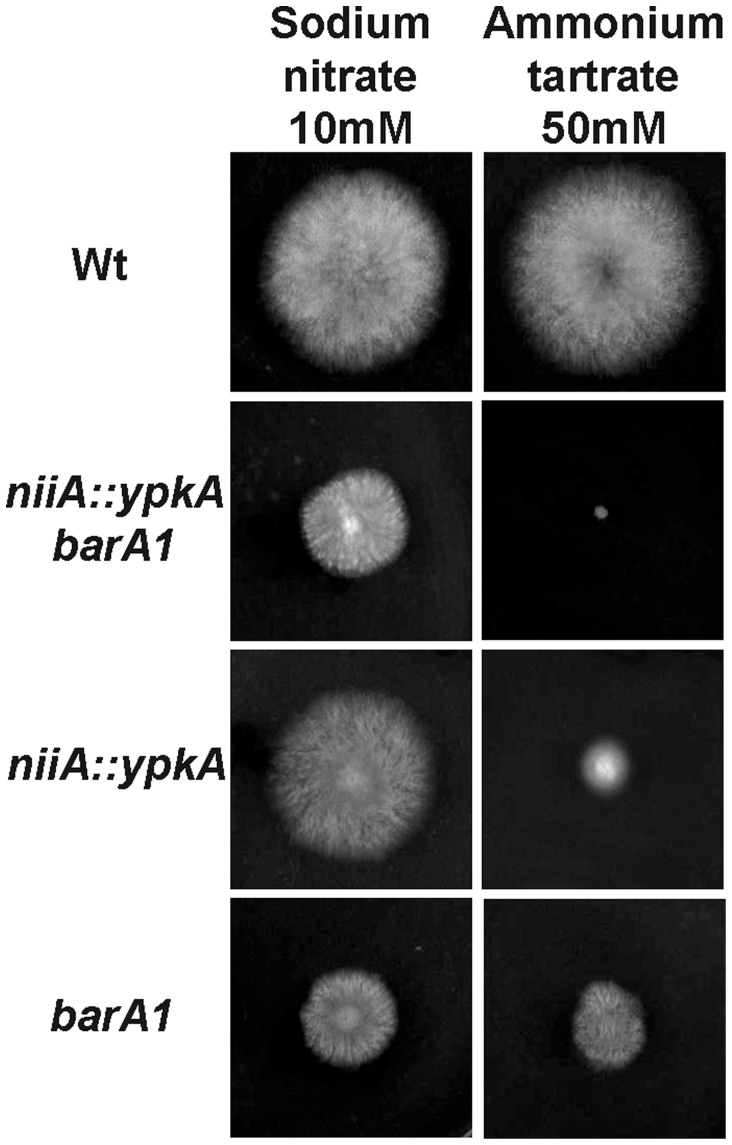
The *A. nidulans* YpkA interacts with BarA. The radial growth of the wild-type, *niiA::ypkA*, *barA1*, and *niiA::ypkA barA1* mutant strains were grown for 72 hours at 37°C on MM+sodium nitrate 10 mM or MM+ammonium tartrate 50 mM (Wt = Wild-type).

Ceramides and sphingoid long-chain bases (LCBs) are precursors for more complex sphingolipids and play distinct signaling roles crucial for cell growth and survival [49]. It has been shown that *A. nidulans* has two ceramide synthases that regulate hyphal morphogenesis and one of them, BarA, is unique to filamentous fungi [50]. Since *YPK1* is important in the sphingolipid-mediated signaling pathway in yeast [5] the interactions between the *niiA::ypkA* and *barA1* mutants was investigated (Figure 9). The *niiA::ypkA barA1* double mutant showed a synthetic lethality phenotype when grown in the presence of ammonium tartrate (Figure 9), suggesting there is a genetic interaction between YpkA and BarA.

### Transcriptional Profiling of Increased and Reduced YpkA Expression

To gain an insight into which genes and pathways were influenced by YpkA, the transcriptional profile of the *A. nidulans alcA::ypkA* strain, cultured under *ypkA* overexpression and repression conditions, was determined. The *alcA::ypkA* mutant was selected instead of the *niiA::ypkA* mutant because it permitted the investigation of genes that are affected by *ypkA* overexpression. The *A. nidulans* wild-type and *alcA::ypkA* mutant strains were grown for 16 and 24 hours in the presence of 4% glucose (repression) or 2% glycerol plus 100 mM threonine (overexpression). Under *ypkA* repression conditions, 186 genes were differentially modulated in at least one time point (85 and 101 genes up and down regulated, respectively, *p*<0.001), while under *ypkA* overexpression conditions 132 genes were differentially modulated in at least one time point (57 and 75 up and down regulated, respectively, *p*<0.001) compared to the wild-type control. Hierarchical clustering of the significant genes sets identified four main up and down regulated clusters under *ypkA* repression and three main up and down regulated clusters under *ypkA* overexpression (Supplementary Figure 1). The microarray data was deposited at https://www.ncbi.nlm.nih.gov/geo/query/acc.cgi?acc=GSE39755, while the significant gene sets are presented in Supplementary Table S1.

**Figure 10:**
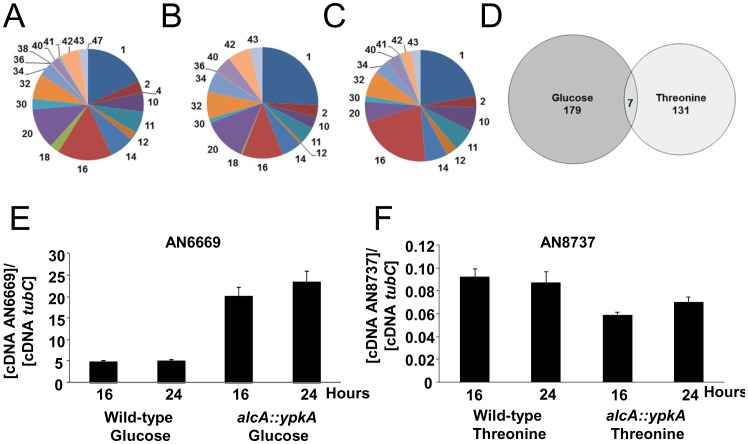
The representation of the MIPS FunCats in the functionally annotated genes in the wild-type *A. nidulans* genome (A) and the genes differentially expressed (*p*<0.001) in the *alcA::ypkA* strain under repression (B) and overexpression (C) conditions when compared to the wild-type strain. The venn diagram demonstrates the limited overlap between the differentially expressed genes under *ypkA* repression and overexpression conditions (D). Real-time RT-PCR for AN6669 and AN8737 genes with increased and decreased mRNA accumulation in the wild-type and *alcA::ypkA* mutant strains grown in glucose 4% or threonine 100 mM, respectively, for 16 and 24 hours.

The differentially expressed genes in the *alcA::ypkA* strain, under the two conditions, were classified into MIPS functional categories (http://mips.helmholtz-muenchen.de/proj/funcatDB/search_main_frame.html) [51]. Within the glucose repressed or threonine induced gene sets no single FunCat was significantly overrepresented compared to the ratio in the genome (Fisher’s test, *p*<0.01; data not shown). Close inspection of gene function revealed conserved themes across the gene sets that correlated with the observed phenotypes under the two conditions including nutrient limitation related metabolism and uptake, cell membrane and wall biosynthesis, protein modification, membrane trafficking and cell cycle (Supplementary Table S2). However, despite having a similar functional profile the majority of the genes within the two data sets were distinct (Figure 10).

Under *ypkA* repression there was a down regulation of genes involved in carbohydrate metabolism including an alcohol dehydrogenase (AN2860) that plays a role in fermentation and a phosphogluconate dehydrogenase (AN6135) that forms part of the pentose phosphate shunt. Simultaneously there was an up regulation of the high affinity glucose sensor *RGT2* (AN6669), sugar transporter *HXT17* (AN1865) plus multiple polysaccharide degrading enzymes (AN5727, AN8421, AN0479, AN9365). Conversely, *ypkA* overexpression caused an up regulation of a pyruvate dehydrogenase kinase (AN10800) that inactivates the pyruvate dehydrogenase complex and the *RGT1* (AN8177) glucose responsive transcription factor, while a down regulation of *SNF3* (AN8737) the low glucose sensor and xylolytic enzymes (AN0942, AN8149). Real-time PCR experiments showed that AN6669 and AN8737 were transcriptionally modulated as observed in the microarray hybridization experiments (Figures 10E–F). The repression of *ypkA* also impacted the nitrogen status of the cell with an up regulation of genes involved in amino acid synthesis (AN1857, AN1858, AN1034) and the down regulation of multiple amino acid permeases (AN2201, AN11211, AN6770, AN2781). Overexpression also resulted in the up regulation of amino acid biosynthesis genes (AN8608, AN8009, AN3928, AN5200). The acquisition of other nutrients was also modulated by *ypkA* expression including the up regulation of inositol and phosphate uptake under *ypkA* repression (AN3781, AN2864), iron (AN0176) and sulfur (AN2200, AN1825) under *ypkA* overexpression, while fatty acid (AN5028, AN7320, AN9408) and potassium transporting ATPase energy metabolism (AN6642) were down regulated by *ypkA* repression.

The influence of *ypkA* also impacted at the protein level including elevated expression post *ypkA* overexpression of the cyclin (AN9500) that interacts with and is phosphorylated by Pho85p cyclin-dependent kinase (Cdk), induced by Gcn4p at level of transcription, and specifically required for Gcn4p degradation and may act as a sensor of the cellular capacity to protein synthesis [52]. Genes involved in protein glycosylation (repression: AN5888, AN2045) and proteolysis (repression: AN6686; overexpression: AN3932, AN8681) were up regulated during *ypkA* repression and overexpression. While there was a down regulation of genes which transcribe GPI anchored proteins (repression: AN11048, AN5033, AN10790, AN4381: overexpression AN6969) and a different set of peptidase (repression and overexpression: AN6438; overexpression: AN8445, AN3959, AN9003).

Genes involved in sterol biosynthesis and cell wall formation were influenced at the transcriptional level by *ypkA* expression, including glucan modifying, phospholipid metabolism and ergosterol biosynthesis enzymes. Under both *ypkA* repression and overexpression there was a greater number of genes involved in these processes which were down regulated (Supplementary Table S2). In addition, genes involved in the establishment of polarized growth and membrane-to-vacuole trafficking were also influenced by *ypkA*. Repression resulted in the up regulation of the voltage-gated calcium channel *CCH1* (AN1168) and a subunit of the ESCRT I complex (AN2521) involved in trafficking vesicles from the plasma membrane to vacuole. Overexpression of *ypkA* caused the down regulation of the ARP2/3 complex (AN8698) involved in the establishment of the actin cortical patch which is found in the polarized regions of cells, while causing the up regulation of a palmitoyltransferase (AN1907) that acts upon SNARE proteins which play a role in the fusion of vesicles with membrane for external or vacuolar secretion. A polyphosphate kinase (AN12017) that plays a role in vacuolar transport and fusion, a vacuolar import protein (AN5157) and a subunit of the exocyst complex involved in secretion (AN6210) were also up regulated under *ypkA* overexpression.

Cell cycle may have also been influenced by the activity of *ypkA*, as a putative transcription factor (AN0273), which is required for septum destruction during cytokinesis, cell polarity and morphogenesis, as well as a protein involved in cell cycle arrest (AN6617) were up regulated under *ypkA* repression. In addition, the down regulation of an arginase (AN6869), which when mutated in *S. cerevisiae* results in the arrest of cell growth in unbudded G1 phase, and the up regulation of an endopeptidase (AN6686) similar to the *S. cerevisiae* Bar1 (not an ortholog of BarA in *A. nidulans*) [53] which when mutated causes hypersensitivity to alpha factor-induced G1 arrest, suggesting that the repression of *ypkA* activity was influencing the successful progression through the cell cycle in *A. nidulans*.

## Discussion

The serum- and glucocorticoid-inducible kinase (SGK) was initially identified in a screen of a cDNA library generated from mammary tumor cells of rats treated with glucocorticoids [1]. The SGK kinase has since been revealed to be expressed in a wide variety of species. The three SGK isoforms that exist in mammals regulate metabolism, transport, transcription and the enzymatic activation of a diverse range of functions such as epithelial transport, excitability, cell proliferation, and apoptosis [54]. The *S. cerevisiae* SGK homologues, Ypk1 and Ypk2 [6], are required for cell growth [55], cell wall integrity [38], [56], while being involved in endocytosis [16], [44], actin polarization [23] and sphingolipid metabolism [57]. Both the *Δypk1* and *Δypk2* deficient strains grew slower than the wild-type strain, while the double *Δypk1 Δypk2* mutant was not viable, with germilings undergoing two or three rounds of cell division prior to arresting growth [55].

The presented study demonstrated that *A. nidulans* has a single *ypk1* homologue with significant similarity to Ypk1 and Ypk2p, which proved to be essential to cell viability. Replacement of the endogenous *ypkA* promoter with two different regulatable promoters, *alcA* and *niiA*, confirmed *ypkA* was an essential gene. The *A. nidulans ypkA* conditional mutants showed that reduced *ypkA* expression caused decreased radial growth, delayed conidial germination, deficient polar axis establishment, intense branching during the late stages of growth, a lack of asexual spores, and a terminal phenotype. These effects were more noticeable after approximately 16 hours of growth, since at this point more than 90% of the germlings showed an increasingly unordered and branched growth pattern, as well as a defect in hyphal elongation.

The spatial control of cell growth involves cell wall synthesis and organization of the cytoskeleton. A polarized cytoskeleton is important for secretion at the hyphal apex and is essential for the establishment and maintenance of polarized growth [38]. The repression of *ypkA* caused CFW and SDS resistance, suggesting modification in the cell wall structure compared to the wild type strain. FITC-conjugated WGA staining demonstrated that the sites of cell wall deposition were mislocalized or absent during *ypkA* repression. The transcriptomic analysis revealed that *ypkA* modulates the transcription of GPI anchored proteins, which are maybe involved with cell wall formation and polarized growth. The fluorescently labeled YpkA protein was distributed throughout cytoplasm and partial co-localized with the microtubules indicating that YpkA may participate in intracellular trafficking and the delivery cell membrane and/or wall constituents to the hyphal apex. Low levels of YpkA make cell wall and perhaps other precursors distributed erroneously along the hyphae.

The sterol composition in animals and yeasts influences the apical localization of proteins. Ergosterol is an important raw material for new cell membranes and low ergosterol concentrations stimulate Ypk1 activity, thus Ypk1 may act as a sensor of ergosterol levels coordinating cell wall synthesis and budding [38], [58]. The lipid rafts are specialized membrane structures, consisting of an aggregation of sphingolipids and ergosterol, that mediate biosynthetic and endocytic processes by anchoring compounds to the plasma membrane. These domains rich in sterol and sphingolipids play an important role in cellular processes including addressing proteins, polarity and signal transduction [31]. The repression of *ypkA* increased sensitivity to lovastatin, an inhibitor of HGM-CoA reductase and sterol synthesis, suggesting there was a lower production of ergosterol when YpkA was reduced. Furthermore, filipin staining showed that ergosterol-rich membrane domains were disorganized upon *ypkA* repression, while *ypkA* was also shown to modulate the transcription of genes involved in sterol biosynthesis. Collectively, these results indicate that YpkA is involved in the proper synthesis and localization of lipid rafts in *A. nidulans*.

In mammals, SGK is activated by the phosphatidylinositol dependent kinase-1 (PDK-1), which responds to increased levels of phosphatidylinositol. The *S. cerevisiae* homologues Pdk1 and Pdk2 are essential and are both involved in the activation of *ypk1* and *ypk2*. Sphingolipids stimulate Pkh1 auto–phosphorylation, while Pkh1 is required for Ypk1 phosphorylation [6], [9]. The *pkh1* mutant showed defects in actin polarization at restrictive temperatures and an inability to initiate the endocytic processes [9]. Phytosphingosine (PHS) generation depends on the concentration of phytoceramides in plasma membrane. Thus, Pkh1-Ypk1 signaling cascade represents a feed-back mechanism which coordinates membrane growth and cell wall expansion [56]. In *S. cerevisiae*, Ypk1 phosphorylation is increased in presence of PHS [5]. The *A. nidulans* Pkh1 homologue *pkhA* was also an essential gene. In an initial attempt to verify if PkhA and YpkA were interacting in *A. nidulans*, a double mutant *niiA::pkhA alcA::ypkA* was constructed and both genes were individually or simultaneously induced or repressed. When *pkhA*, *ypkA* or both genes were repressed, the mutant displayed a similar growth phenotype, suggesting that the ypkA gene is not directly downstream of *pkhA* or epistatic to *pkhA*. These results strongly indicate that in *A. nidulans*, differently from what is observed in *S. cerevisiae*, *ypkA* and *pkhA* are genetically independent or in parallel. It remains to be investigated which pathways are responsible for YpkA activation.

The activity of the mammalian growth factor-regulated PDK-1 is induced by an increased level of phosphatidylinositol. Apart from SGK, PDK1 also phosphorylates other kinases including PKB/Akt, p70 and PKC isoforms. The activation of PKB by PDK1 occurs in presence of vesicles containing phosphatidylinositol tri-phosphate, and results from the interaction of this second messenger with the PKB PH domains. The presented transcriptome analysis of *ypkA* repressed *A. nidulans* demonstrated the increased expression of genes that regulate the uptake of inositol and phosphate, possibly representing an attempt to compensate for the lack of the signaling cascade triggered by the PkhA-YpkA cascade.

Besides the internalization of nutrient, endocytosis allows eukaryotic cells to recycle components of the plasma membrane, regulating the expression of cell signaling receptors and maintaining the proper supply of membrane precursors and removal of excess material thus supporting hyphal growth [45]. The vesicle supply center at the hyphal apex, called the Spitzenkörper, is essential for filamentous growth and determination polarization, performing a role in both endo- and exocytosis [59]. In *S. cerevisiae*, the Pkh–Ypk1 kinase cascade is an important regulatory component of the endocytic machinery [44]. In the presented study, FM4-64 staining was used to monitor the the vacuolar system and endomembranes in *A. nidulans* [31]. During *ypkA* repression the Spitzenkörper was absent and FM4-64 uptake was delayed. Additionally, the transcriptomic analysis of *ypkA* repression revealed the modulation of genes involved with vacuolar trafficking. Accordingly, in *A. nidulans* YpkA was required for proper function of the endocytic machinery. Furthermore, in *S. cerevisiae* Ypk1 affects fatty-acid uptake and thus energy homeostasis via regulating endocytosis [34]. During *ypkA* repression in *A. nidulans*, genes involved in fatty acid and potassium transporting ATPase energy metabolism were down regulated suggesting that YpkA affects the uptake of fatty acids.

*BarA* is the *A. nidulans* homologue of the *S. cerevisiae Lag1* acyl-CoA-dependent ceramide synthase, which catalyzes the condensation of phytosphingosine with a fatty acyl-CoA to form phytoceramide. The *barA* null mutant showed heat sensitivity, a delay in the emergence of germ tubes and fails to maintain a stable axis of polarity and absence of filipin staining at hyphal tips [50]. These phenotypic defects were similar to those observed when sphingolipid biosynthesis was blocked [60]. This appeared similar to the phenotype observed upon *ypkA* repression, while in the absence of *barA*, *ypkA* repression was lethal to the cell viability. Therefore there appears to be a genetic interaction between *ypkA*, *barA*, and the sphingolipid synthesis. Ceramides and sphingoid long-chain bases (LCBs) are precursors to more complex sphingolipids and play distinct signaling roles crucial for cell growth and survival [49]. LCBs can induce cellular responses during heat stress [61]. In *S. cerevisiae*, Ypk1 activates the first enzyme in the sphingolipid biosynthesis pathway through the control of the Orm protein activity [21]. Recent studies reported that Ypk1 regulates the sphingolipid homeostasis during heat stress through Orm protein phosphorylation [62]. A similar mechanism may occur in *A. nidulans*, as low levels of YpkA increased the susceptibility of the cells to restrictive temperatures, probably due to the absence of key elements of the cellular structure.

In *S. cerevisiae*, Ypk1 is rapidly degraded when nitrogen is absent leading to decreased levels of eIF4G and the disruption of protein translation [24]. *A. nidulans* genes involved with protein glycosylation, proteolysis and a set of peptidases were repressed or overexpressed when *ypkA* expression was modulated. Furthermore, genes involved in amino acid transport and metabolism were modulated under both repressing and inducing conditions, possibly in an attempt to restore the normal levels of nitrogen within the cell.

Collectively this investigation suggests that in the filamentous fungus *A. nidulans* YpkA acts as a sensor and an effector protein coordinating the maintenance and expansion of the cell membrane and wall. Depending on the stimulus, YpkA is possibly phosphorylated by PkhA. Subsequently, the active YpkA regulates the synthesis and transport of ergosterol, amino phospholipids and shingolipids, which are packed into lipid rafts, to the hyphal apex permitting polarized growth.

## Materials and Methods

### Strains, Media and Culture Methods

The *A. nidulans* strains used are described in Supplementary Table S3. The media used were of two basic types, complete or minimal. The complete media comprised the following three variants: YAG (2% w/v glucose, 0.5% w/v yeast extract, 2% w/v agar, trace elements), YUU (YAG supplemented with 1.2 g/liter [each] of uracil and uridine), and liquid YG or YG+UU medium with the same composition (but without agar). The minimal media were a modified minimal medium (MM; 1% w/v glucose, original high-nitrate salts, trace elements, 2% w/v agar, pH 6.5). Expression of the *ypkA* gene under the control of *alcA* promoter was regulated by carbon source: repression on glucose 4% w/v, derepression on glycerol, and induction on ethanol or threonine. Therefore, MM-G and MM-E were identical to MM, except that glycerol (2% v/v) and/or ethanol (2% v/v) or threonine (100 mM) were used, respectively, in place of glucose as the sole carbon source. Trace elements, vitamins, and nitrate salts were included as described by [63]. Strains were grown at 37°C unless indicated otherwise. Expression of *ypkA* gene under the control of *niiA* promoter was regulated by nitrate source: repression on a modified minimal medium (AMM) (1% w/v glucose, 2% w/v agar) plus ammonium tartarate 50 mM and induction on AMM plus sodium nitrate 10 mM.

### Molecular Techniques

Standard genetic techniques for *A. nidulans* were used for all strain constructions and genetic transformation [63], [64]. DNA manipulations were performed according to [65]. All PCR reactions were performed using Platinum Taq DNA Polymerase High Fidelity (Invitrogen). The primers used in this work are listed on Supplementary Table S4. For the construction of the *alcA::ypkA* strain, the *ypkA* encoding region was cloned into the pMCB17apx vector downstream to the *alcA* promoter. This construction was transformed into *A. nidulans* to replace the endogenous *ypkA* promoter yielding the strain *alcA::ypkA*. The transformants were confirmed by PCR. The *ΔypkA* deletion cassette was constructed by amplifying 1.5-kb region either side of the ORF. The *A. fumigatus pyroA* gene, which localized in the center of the cassette, acted as a selectable marker by enabling the transformed cells to grow on media without addition of pyridoxine and was amplified from *A.fumigatus* CEA17 strain. Transformants were confirmed by PCR using the heterokaryon rescue technique described by Osmani *et al.* [39]. The cassette *niiA::ypkA* was also constructed by PCR-mediated technique. About 1.5-kb region on both 5′ and 3′-ends of the ORF were selected for primer design. The *pyrG::niiA* fragment was amplified from the *niiA::pikA* strain. The transformants were confirmed by Southern blot analysis (Supplementary Figure S2).

The construction of the YpkA::GFP fusion gene was performed according to Colot *et al*. [66]. Briefly, 1.5-kb regions either side of the *ypkA* ORF were selected for primer design. The primers amplified the 5′-UTR flanking region plus the *ypkA* ORF and the 3′-UTR flanking region. Both fragments were PCR amplified from genomic DNA of the *A. nidulans* A4 strain. The *pyrG* gene, used as a selective marker for uridine and uracil prototrophy was amplified from the pCDA21 plasmid, and GFP gene was amplified from the pMCB17apx plasmid. The external primers possessed cohesive ends that matched the pRS426 vector used for *in vivo* recombination in *S. cerevisiae*. The pRS426 vector was double digested with *Eco*RI and *Bam*HI for linearization and the *S. cerevisiae* strain SC9721 was transformed with these four DNA fragments using the lithium acetate method [67]. The DNA of the *S. cerevisiae* transformants was extracted by the method described by Goldman *et al.* [68], dialysed and transformed by electroporation into the *Escherichia coli* strain DH10B, in order to rescue the pRS426 plasmid harboring the cassettes.

All the cassettes were PCR-amplified and used for transformation of *A. nidulans* according to the procedure of OSMANI *et al.* [64]. Transformants were scored for their ability to grow on minimal medium.

### Staining and Microscopy

For germling nuclear and septum staining, conidia were inoculated onto coverslips. After incubation under the appropriate conditions for each experiment, coverslips with the adherent germlings were fixed (3.7% formaldehyde, 50 mm sodium phosphate buffer pH 7.0, 0.2% Triton X-100) for 30 min at room temperature, rinsed with PBS buffer (140 mm NaCl, 2 mm KCl, 10 mm NaHPO4, 1.8 mm KH_2_PO4, pH 7.4) and incubated for 5 min in a solution with 100 ng/ml of DAPI (Sigma Chemical, St. Louis) and/or 100 ng/ml of calcofluor (fluorescent brightener, Sigma Chemical). After incubation with these dyes, coverslips were washed with PBS for 10 min at room temperature and then finally rinsed with distilled water and mounted for microscopical examination. For cell imaging of YpkA protein fused to GFP, conidiospores were grown in glass-bottom dishes (Mattek, Ashland, MA) at 30°C. Germlings were fixed for 10 min in a fixative solution containing 1× PBS, 5% DMSO, 3.7% formaldehyde, and 10% methanol. The nucleus was DAPI stained as described above. For lectin staining, coverslips were incubated in prewarmed media containing 5 µg/ml FITC-conjugated wheat germ agglutinin (WGA) for 5 min and then fixed, washed, and mounted. For lipid raft staining, the cells were stained with filipin (25 mg ml^−1^) for 5 min and then analysed. In order to monitor the establishment of polarization, conidia were incubated in the appropriate experimental conditions and samples taken at 2 hour intervals over 16 hours. A conidium was counted as polarized if it possessed a germ tube readily detectable as small protuberances on the spherical shape of conidium surface.

Slides were viewed with a Carl Zeiss (Jena, Germany) microscope using 100× magnification oil immersion objective (EC Plan-Neofluar, NA 1.3) equipped with a 100 W HBO mercury lamp. Phase contrast for the brightfield images and fluorescent images were captured with an AxioCam camera (Carl Zeiss), processed using the AxioVision software version 3.1 and saved as TIFF files. Further processing was performed using Adobe Photoshop 7.0 (Adobe Systems Incorporated, CA).

### RNA Extraction, cDNA Synthesis and Real-time PCR Reactions

After harvesting, mycelia were disrupted by grinding under liquid nitrogen, and total RNA was extracted using Trizol (Invitrogen, USA). RNA (10 µg) from each treatment was fractionated in 2.2 M formaldehyde, 1.2% agarose gel, stained with ethidium bromide, and visualized with UV-light in order to check RNA integrity. The samples were submitted to RNAse-free DNAse treatment as previously described [69], purified with RNeasy® Mini Kit (Qiagen), and then quantified in the NanoDrop® 2000 Thermo Scientific (Uniscience).

All the RT-qPCR reactions were performed using an ABI 7500 Fast Real-Time PCR System (Applied Biosystems, USA) and Taq-Man™ Universal PCR Master Mix kit (Applied Biosystems, USA). The reactions and calculations were performed according to Semighini *et al.* [69]. The primers and Lux™ fluorescent probes (Invitrogen, USA) used in this work are described in Supplementary Table S4.

### Microarray Slides Construction and Gene Expression Methods

To construct the microarray slides the Agilent E-array software tool was used (available at https://earray.chem.agilent.com/earray/). Briefly, we uploaded gene sequences representing the whole *A. nidulans* A4 gene sequences. The ORF number was carefully validated by comparing the sequences deposited in three databanks [CADRE (The Central Aspergillus Resource); AspGD (Aspergillus Genome Database) and BROAD Institute] aiming to identify and validate the sequences for probe design. 11,251 ORFs were submitted to Agilent E-array. Based on some quality parameter implemented in Aglilent E-array (such as sequences with high score for cross-hybridization potential throughout the genome and sequences which no appropriate regions could be found as target to be represented in the slides), 11,143 probes were designed form the uploaded sequence of A4 strain. These probes were represented three-four times in the microarray slides and the annotation based on [1], [2] was used to generate the annotation file used in the analysis. Therefore, the microarray slides comprise 45,220 features representing 1,417 Agilent internal controls and 800 internal controls which represent 80 randomly chosen *A. nidulans* ORFs (ten times replicated each).

The gene expression analysis used in this work was carried out using custom-designed oligonucleotides slides (4×44 K microarray) from Agilent Technologies™, based on *A. nidulans* genome annotation publicly available. After RNA isolation and purification, as described above, the samples were labeled with Cy-3 or Cy-5-dUTP using the Two-Color Microarray-based gene expression analysis (Quick Amp Labeling Kit, Agilent Technologies™, USA) following manufacturer’s protocol. Initially, 5µg of total RNA was incubated with Agilent™ RNA Spike-In controls probes (RNA Spike A or B mix). Prior to labeling, synthesis of cDNA was carried out incubating the samples with 1.2 µL T7 promoter primer, and nuclease-free water in an appropriate volume. The template and primer were denatured by incubating the reaction at 65°C in a circulating water bath for 10 minutes, and after the reactions were placed on ice for 5 min. To the samples were added cDNA Master Mix (4 µL 5X First Strand Buffer, 2 µL 0.1 M DTT, 1 µL 10 mM dNTP mix, 1 µL MMLV-RT and 0.5 µL RNaseOut), and the mixture was incubated at 40°C in a circulating water bath for 2 hours. After, the samples were moved to a 65°C circulating water bath and incubated for 15 minutes. cRNA amplification and labeling were performed by adding to the samples the Agilent™ Transcription Master Mix (20 µL 4× Transcription Buffer, 6 µL 0.1 M DTT, 8 µL NTP mix, 6.4 µL 50% PEG, 0.5 µL RNase OUT, 0.6 µL inorganic pyrophosphatase, 0.8 µL T7 RNA Polymerase, 2.4 µL Cyanine 3-CTP to control samples, or cyanine 5-CTP to treated samples, and 15.3 µL nuclease-free water), and incubating the mixtures in a circulating water bath at 40°C for 2 hours. The labeled cRNA was purified using RNeasy® Mini Kit (Qiagen), and then quantified in the NanoDrop® 2000 Thermo Scientific (Uniscience).

For the hybridization, 825 ng of each labeled cRNA was mixed with Agilent™ Fragmentation Mix (11 µL 10× Blocking agent, 2.2 µL 25X fragmentation buffer, and nuclease-free water to bring the volume to 52.8 µL), and incubated at 60°C for exactly 30 minutes to fragment RNA. The fragmentation was interrupted by adding 55 µL of 2X GE Hybridization Buffer HI-RPM. Finally, 100 µL of sample was placed down onto the microarray slide, which was mounted into the Agilent™ Microarray Hybridization Chamber Kit. The hybridization was carried out in an oven (Agilent G2545A Hybridization Oven) set to 65°C for 17 hours. After, microarray slides were washed according to Agilent’s instruction and scanned using GenePix® 4000B microarray scanner (Molecular Devices, USA).

### Gene Expression Analysis

The extraction of data from TIFF images generated through scanning of microarray slides were performed by using Agilent Feature Extraction (FE) Software version 9.5.3.1 (Agilent Technologies, USA), using Linear Lowess algorithm to obtain background subtracted and normalized intensity values. The dye-normalyzed values generated in the Feature Extraction data files were used to upload the software Express Converter. (version 2.1, TM4 platform available at http://www.tm4.org/utilities.html), which conveniently converts the Agilent file format to mev (multi experiment viewer) file format compatible to the TM4 softwares for microarray analysis (available at http://www.tm4.org/). The mev files were then uploaded in the MIDAS software (TM4 platform), where the resulting data were averaged from replicated genes on each array, from three biological replicates of each treatment. The generated mev files were finally analyzed by using TIGR MeV (TM4 platform, Multi Experiment Viewer, available at http://www.tigr.org/software/microarray.shtml), where differentially expressed genes was statistically identified using one-class *t* test (p>0.01). Significantly different genes were those whose mean log_2_ expression ratio over all included samples was statistically different from 0, which indicates the absence of gene modulation.

## Supporting Information

Supplementary Figure 1 The heat map and centroid plot of the ten hierarchal clusters of genes identified as being differentially expressed (p<0.001) in the alcA::ypkA strain under repression (G1-10) and overexpression (T1-10) conditions when compared to the wild-type strain.
(PDF)

Table S1 Significant gene sets.
(XLSX)

Table S2 Log2 fold change compared to wild-type.
(XLSX)

Table S3 Strains used in this work.
(DOCX)

Table S4 Primers and probes used in this work.
(DOCX)
