# Supplementary material for: Functional Characterization of Aspergillus nidulans ypkA, a Homologue of the Mammalian Kinase SGK
Source: PLoS One. 2013 Mar 5;8(3):e57630. doi: 10.1371/journal.pone.0057630 (PMC3589345; doi:10.1371/journal.pone.0057630)
Supplement: Supplementary Figure 1 — The heat map and centroid plot of the ten hierarchal clusters of genes identified as being differentially expressed (p<0.001) in the alcA::ypkA strain under repression (G1-10) and overexpression (T1-10) conditions when compared to the wild-type strain. (PDF) [file pone.0057630.s001.pdf]

Supplementary Figure 1 – The heat map and centroid plot of the ten hierarchal clusters of genes identified as being differentially expressed (p<0.001) in the *alcA::ypkA* strain under repression (G1-10) and overexpression (T1-10) conditions when compared to the wild-type strain.

Cluster G1

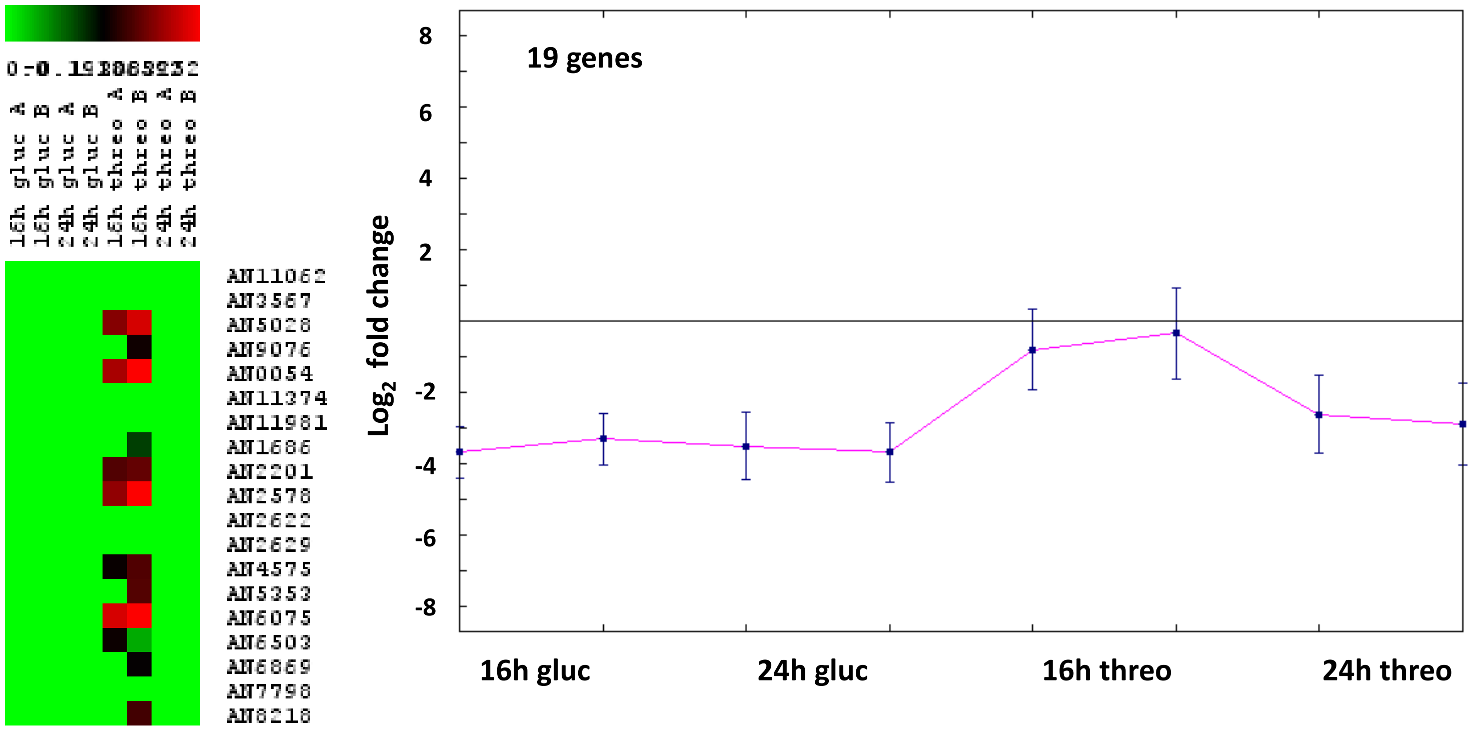

Cluster G2

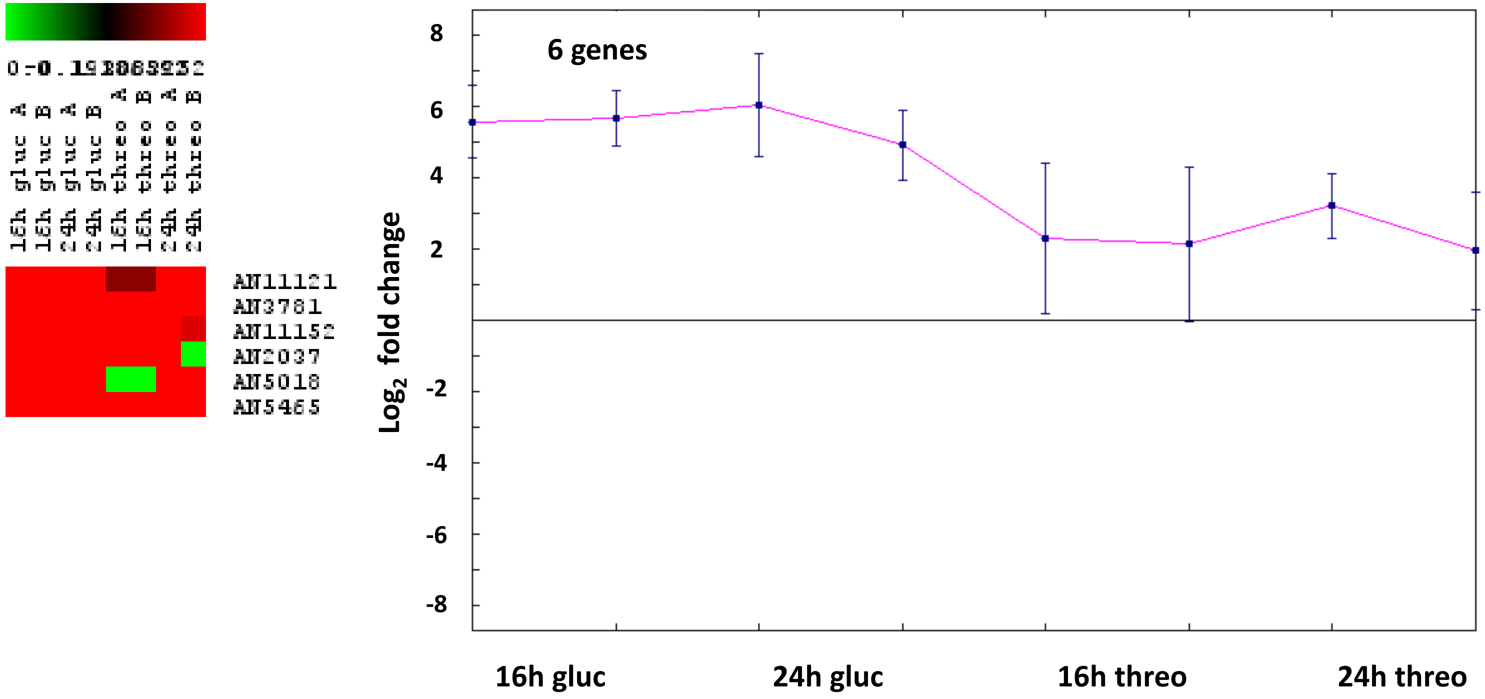
16h gluc A
16h gluc B
24h gluc A
24h gluc B
16h threo A
16h threo B
24h threo A
24h threo B
AN11121
AN3781
AN11152
AN2037
AN5018
AN5465
6 genes
Log2 fold change
8
6
4
2
-2
-4
-6
-8
16h gluc
24h gluc
16h threo
24h threo

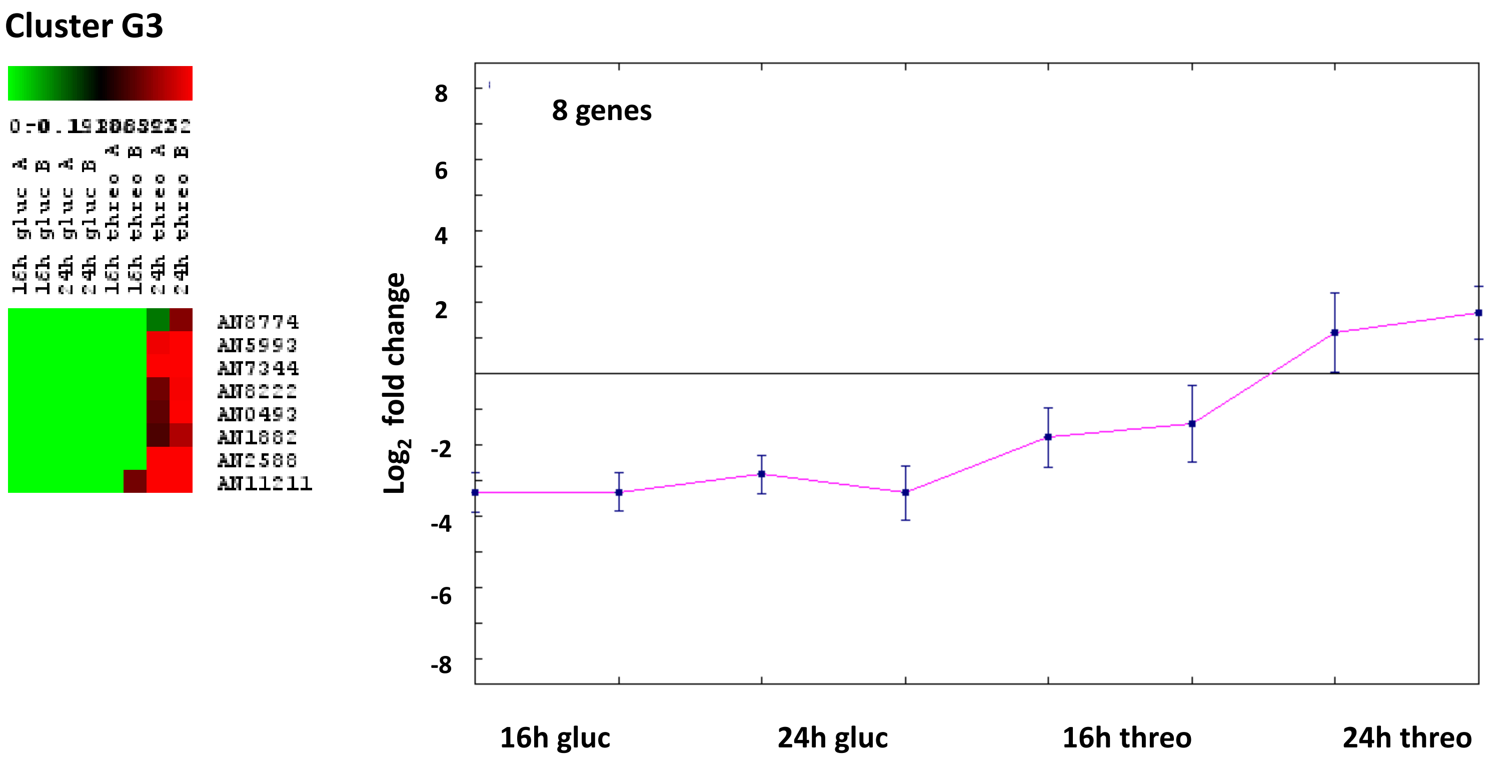
Cluster G3
0 -0.19 ... 2
16h gluc A
16h gluc B
24h gluc A
24h gluc B
16h threo A
16h threo B
24h threo A
24h threo B
AN8774
AN5993
AN7344
AN8222
AN0493
AN1882
AN2588
AN11211
8 genes
Log2 fold change
8
6
4
2
-2
-4
-6
-8
16h gluc
24h gluc
16h threo
24h threo

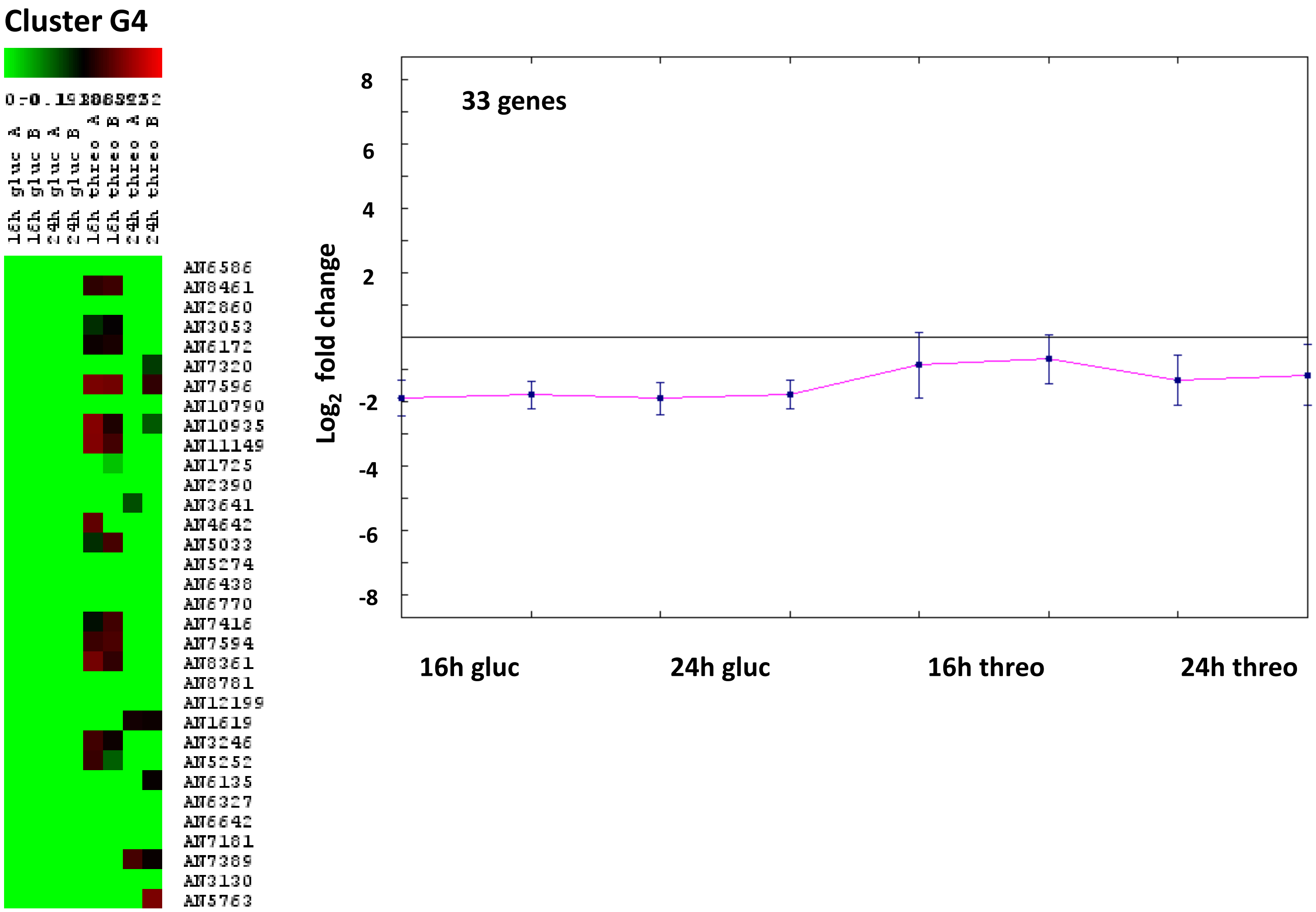
Cluster G4
16h gluc A
16h gluc B
24h gluc A
24h gluc B
16h threo A
16h threo B
24h threo A
24h threo B
AN6586
AN8461
AN2860
AN3053
AN6172
AN7320
AN7596
AN10790
AN10935
AN11149
AN1725
AN2390
AN3641
AN4642
AN5033
AN5274
AN6438
AN6770
AN7416
AN7594
AN8361
AN8781
AN12199
AN1619
AN3246
AN5252
AN6135
AN6327
AN6642
AN7181
AN7389
AN3130
AN5763
33 genes
Log2 fold change
8
6
4
2
-2
-4
-6
-8
16h gluc
24h gluc
16h threo
24h threo

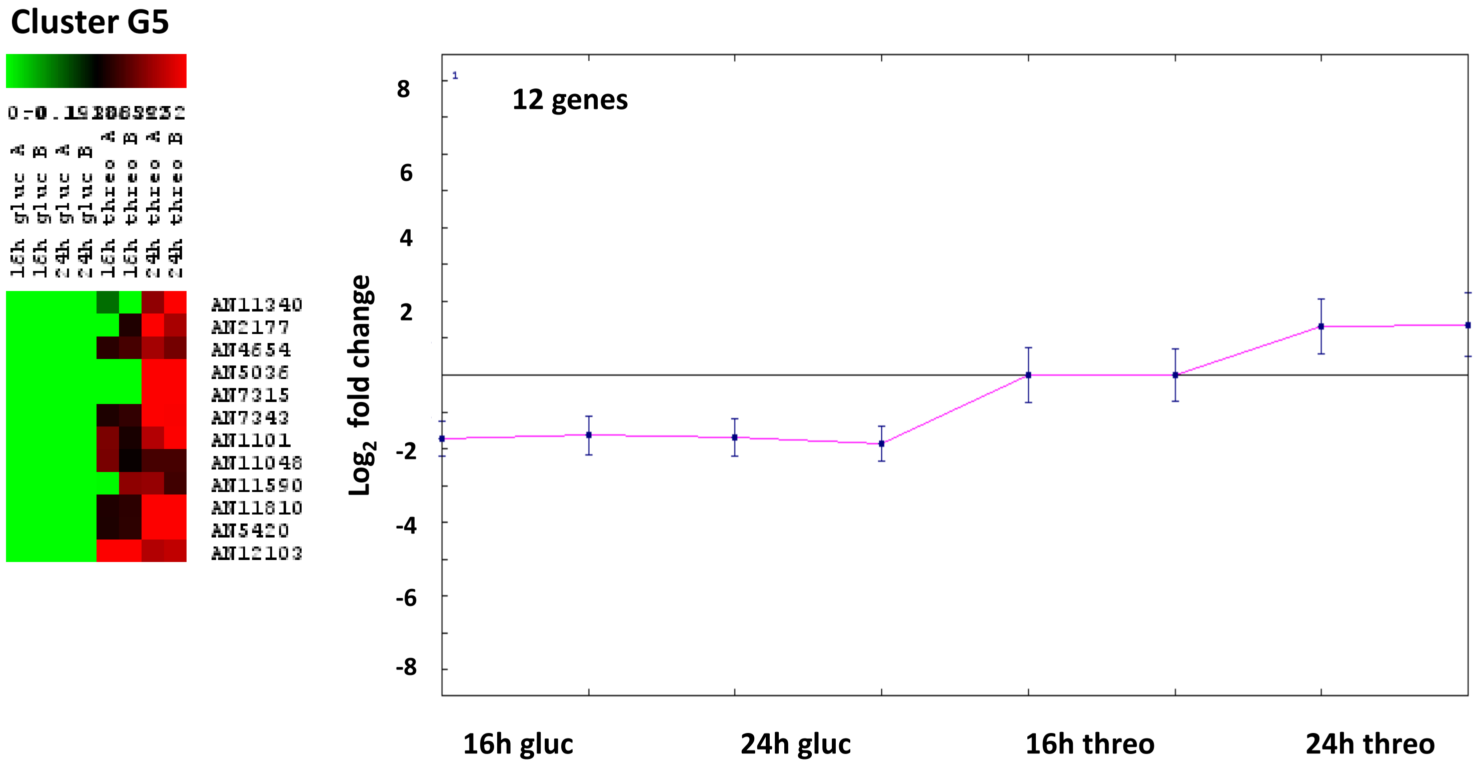

Cluster G5
16h gluc A
16h gluc B
24h gluc A
24h gluc B
16h threo A
16h threo B
24h threo A
24h threo B
AN11340
AN2177
AN4654
AN5036
AN7315
AN7343
AN1101
AN11048
AN11590
AN11810
AN5420
AN12103
12 genes
Log2 fold change
8
6
4
2
-2
-4
-6
-8
16h gluc
24h gluc
16h threo
24h threo

Cluster G6

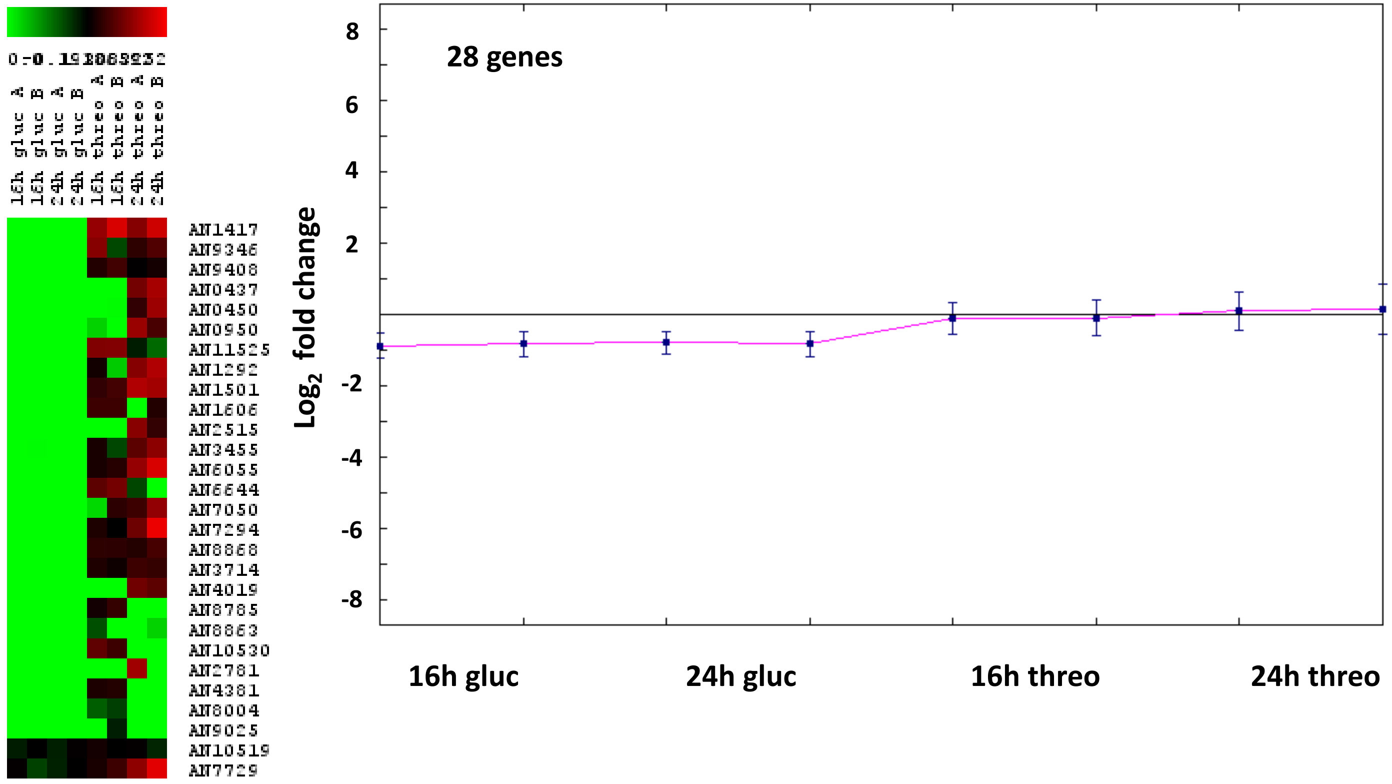
16h gluc A
16h gluc B
24h gluc A
24h gluc B
16h threo A
16h threo B
24h threo A
24h threo B
AN1417
AN9346
AN9408
AN0437
AN0450
AN0950
AN11525
AN1292
AN1501
AN1606
AN2515
AN3455
AN6055
AN6644
AN7050
AN7294
AN8868
AN3714
AN4019
AN8785
AN8863
AN10530
AN2781
AN4381
AN8004
AN9025
AN10519
AN7729
28 genes
Log2 fold change
8
6
4
2
-2
-4
-6
-8
16h gluc
24h gluc
16h threo
24h threo

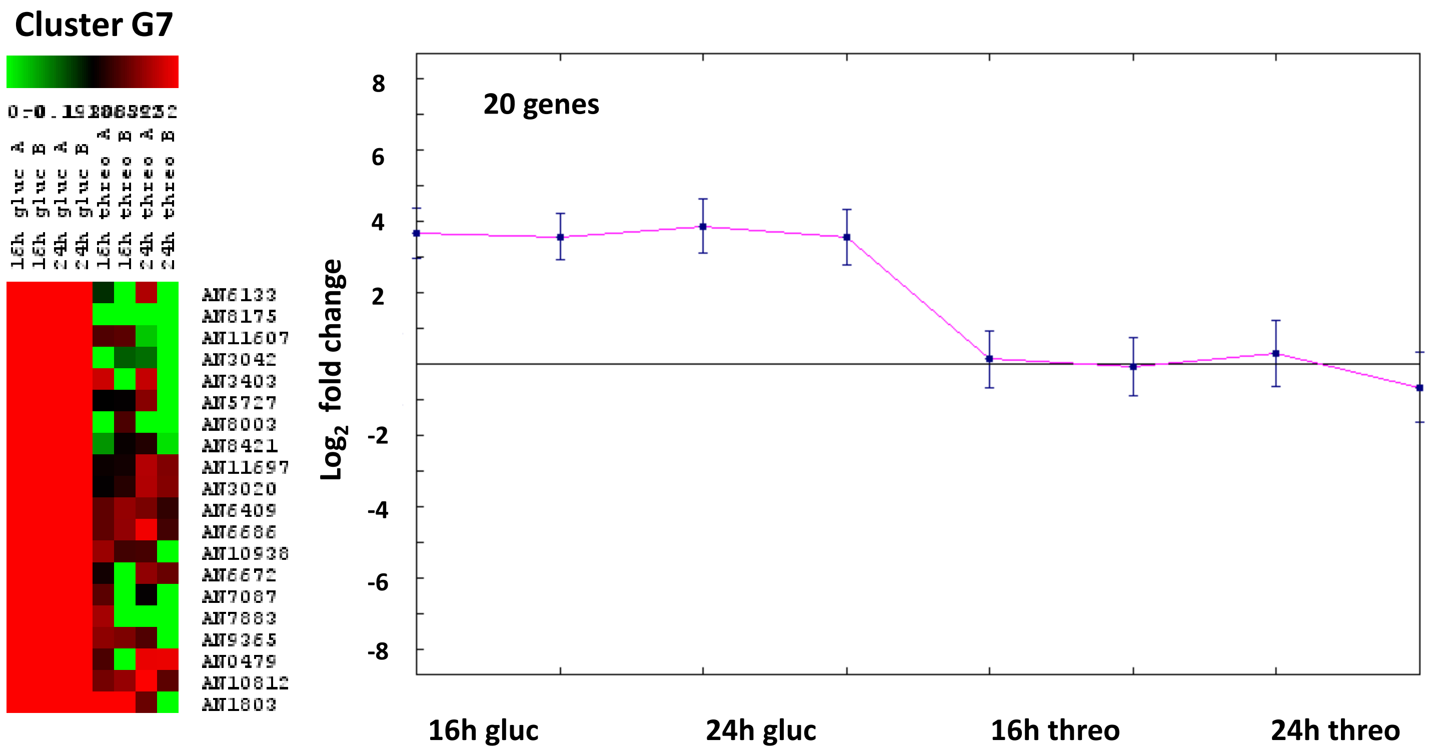
Cluster G7
16h gluc A
16h gluc B
24h gluc A
24h gluc B
16h threo A
16h threo B
24h threo A
24h threo B
AN6133
AN8175
AN11607
AN3042
AN3403
AN5727
AN8003
AN8421
AN11697
AN3020
AN6409
AN6686
AN10938
AN6672
AN7087
AN7883
AN9365
AN0479
AN10812
AN1803
20 genes
8
6
4
2
-2
-4
-6
-8
Log_2 fold change
16h gluc
24h gluc
16h threo
24h threo

Cluster G8

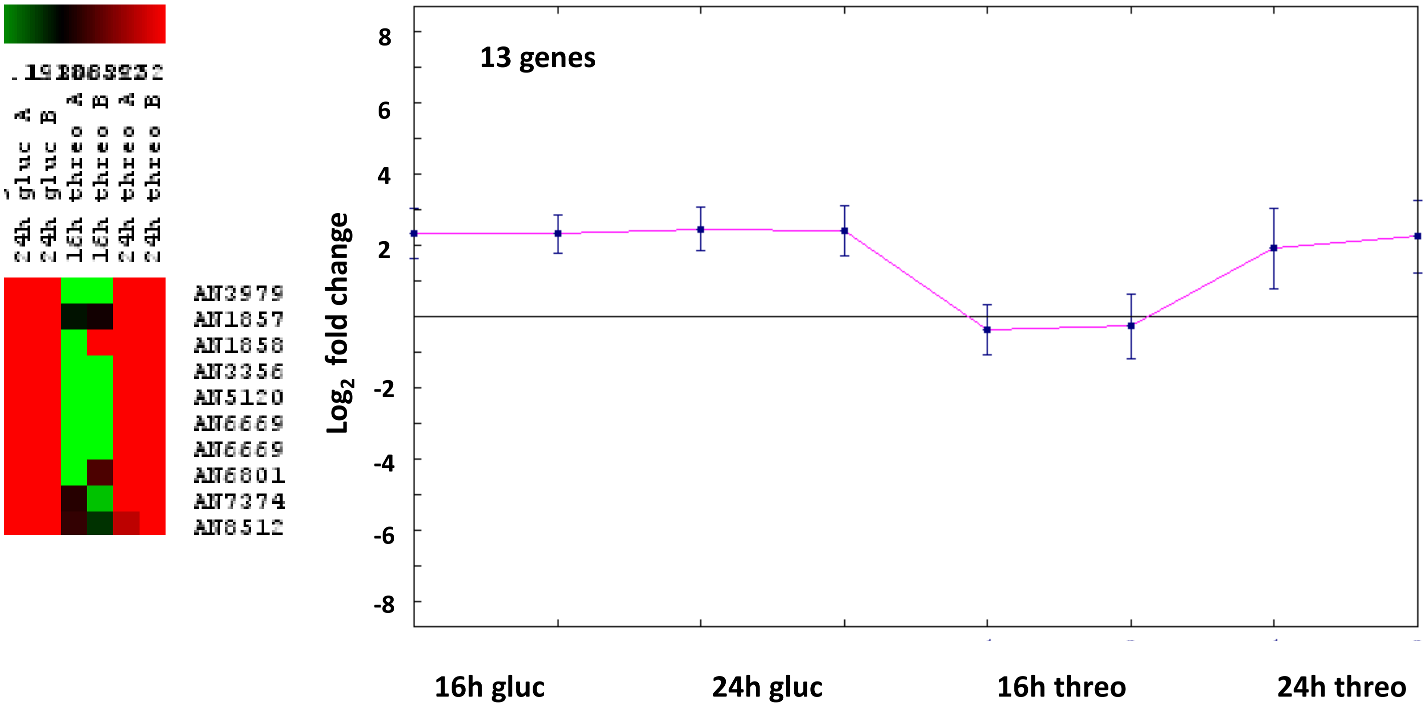

24h gluc A
24h gluc B
16h threo A
16h threo B
24h threo A
24h threo B
AN3979
AN1857
AN1858
AN3356
AN5120
AN6669
AN6669
AN6801
AN7374
AN8512
13 genes
Log2 fold change
8
6
4
2
-2
-4
-6
-8
16h gluc
24h gluc
16h threo
24h threo

Cluster G9

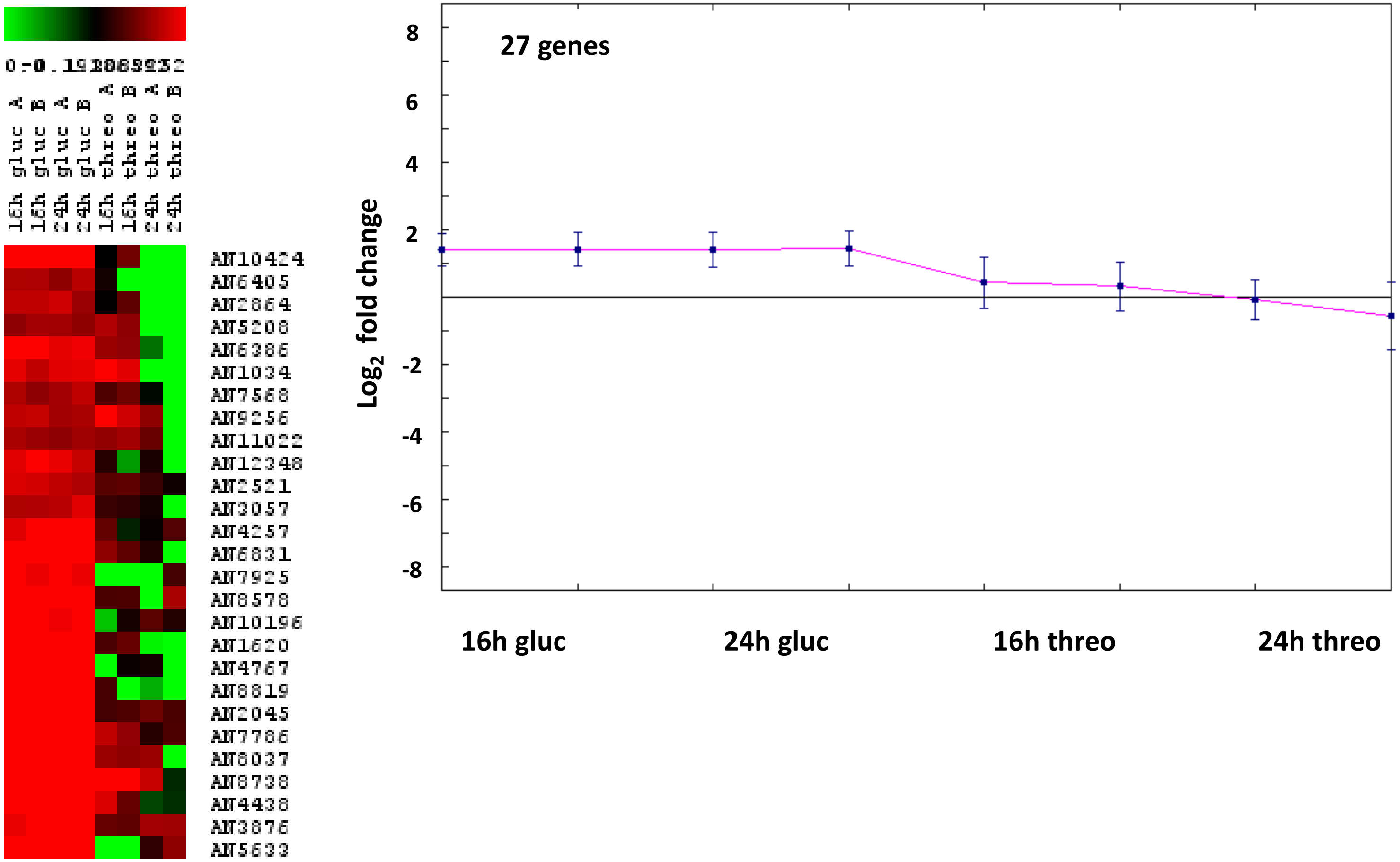
16h gluc A
16h gluc B
24h gluc A
24h gluc B
16h threo A
16h threo B
24h threo A
24h threo B
AN10424
AN6405
AN2864
AN5208
AN6386
AN1034
AN7568
AN9256
AN11022
AN12348
AN2521
AN3057
AN4257
AN6831
AN7925
AN8578
AN10196
AN1620
AN4767
AN8819
AN2045
AN7786
AN8037
AN8738
AN4438
AN3876
AN5633
27 genes
Log2 fold change
8
6
4
2
-2
-4
-6
-8
16h gluc
24h gluc
16h threo
24h threo

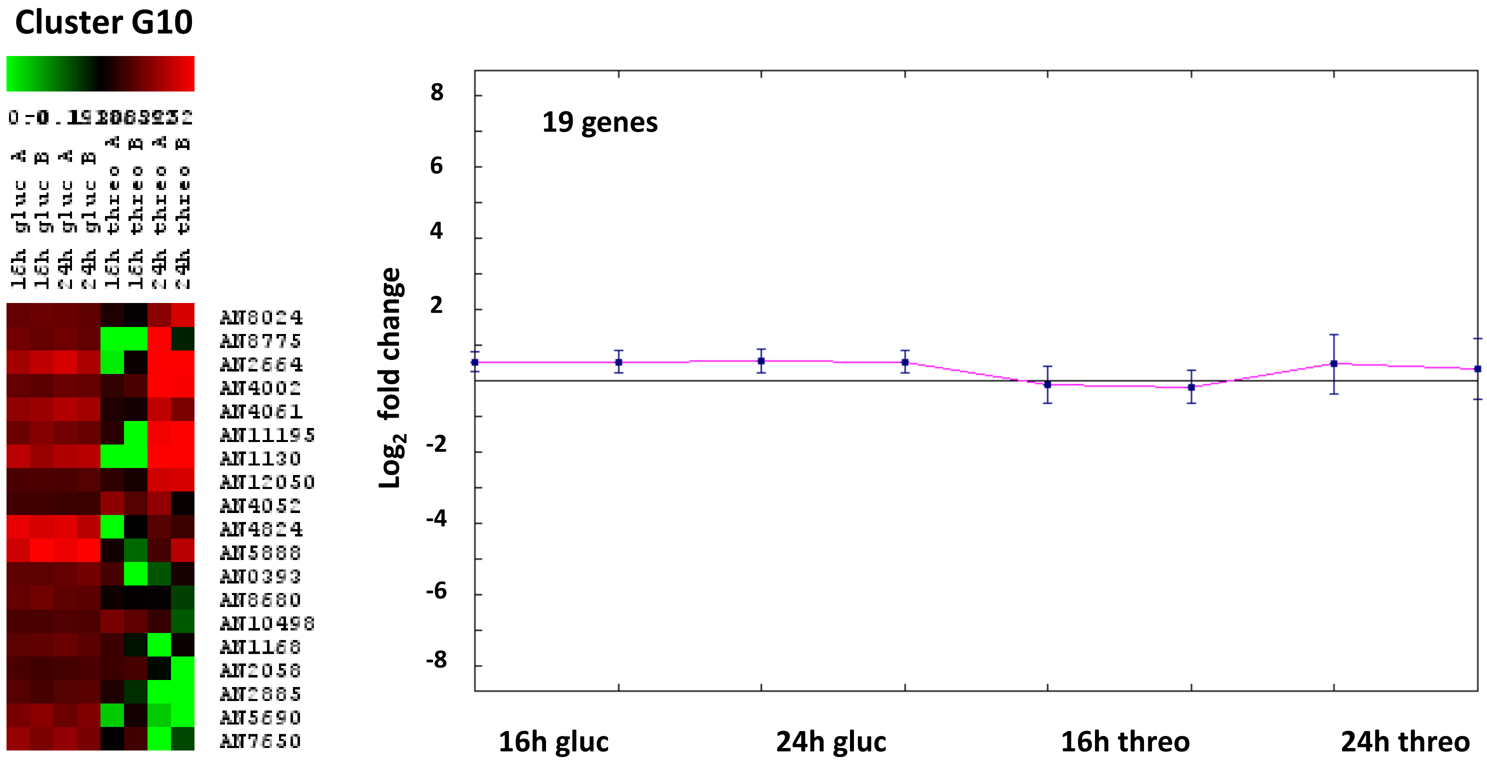
Cluster G10
16h gluc A
16h gluc B
24h gluc A
24h gluc B
16h threo A
16h threo B
24h threo A
24h threo B
AN8024
AN8775
AN2664
AN4002
AN4061
AN11195
AN1130
AN12050
AN4052
AN4824
AN5888
AN0393
AN8680
AN10498
AN1168
AN2058
AN2885
AN5690
AN7650
19 genes
Log_2 fold change
8
6
4
2
-2
-4
-6
-8
16h gluc
24h gluc
16h threo
24h threo

Cluster T1

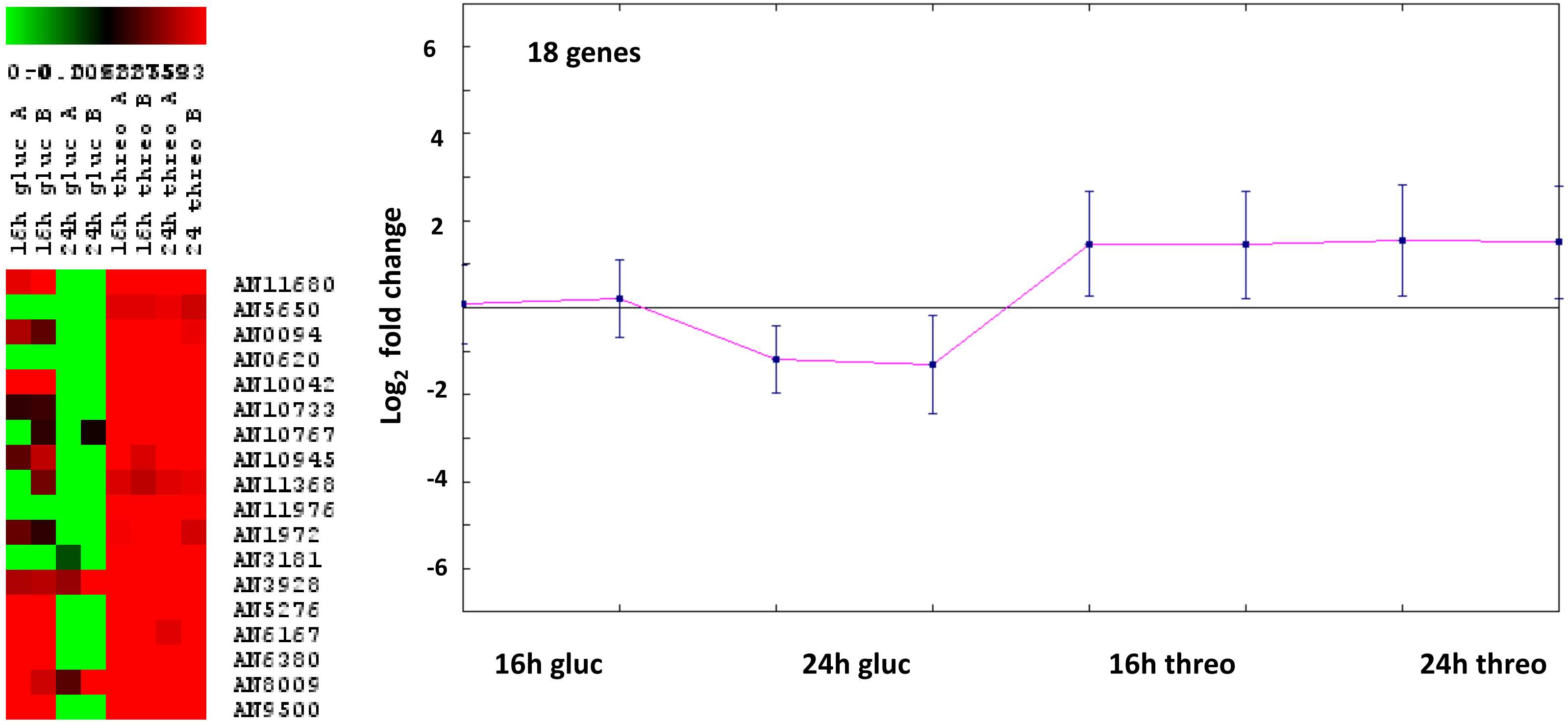
16h gluc A
16h gluc B
24h gluc A
24h gluc B
16h threo A
16h threo B
24h threo A
24 threo B
AN11680
AN5650
AN0094
AN0620
AN10042
AN10733
AN10767
AN10945
AN11368
AN11976
AN1972
AN3181
AN3928
AN5276
AN6167
AN6380
AN8009
AN9500
18 genes
Log_2 fold change
6
4
2
-2
-4
-6
16h gluc
24h gluc
16h threo
24h threo

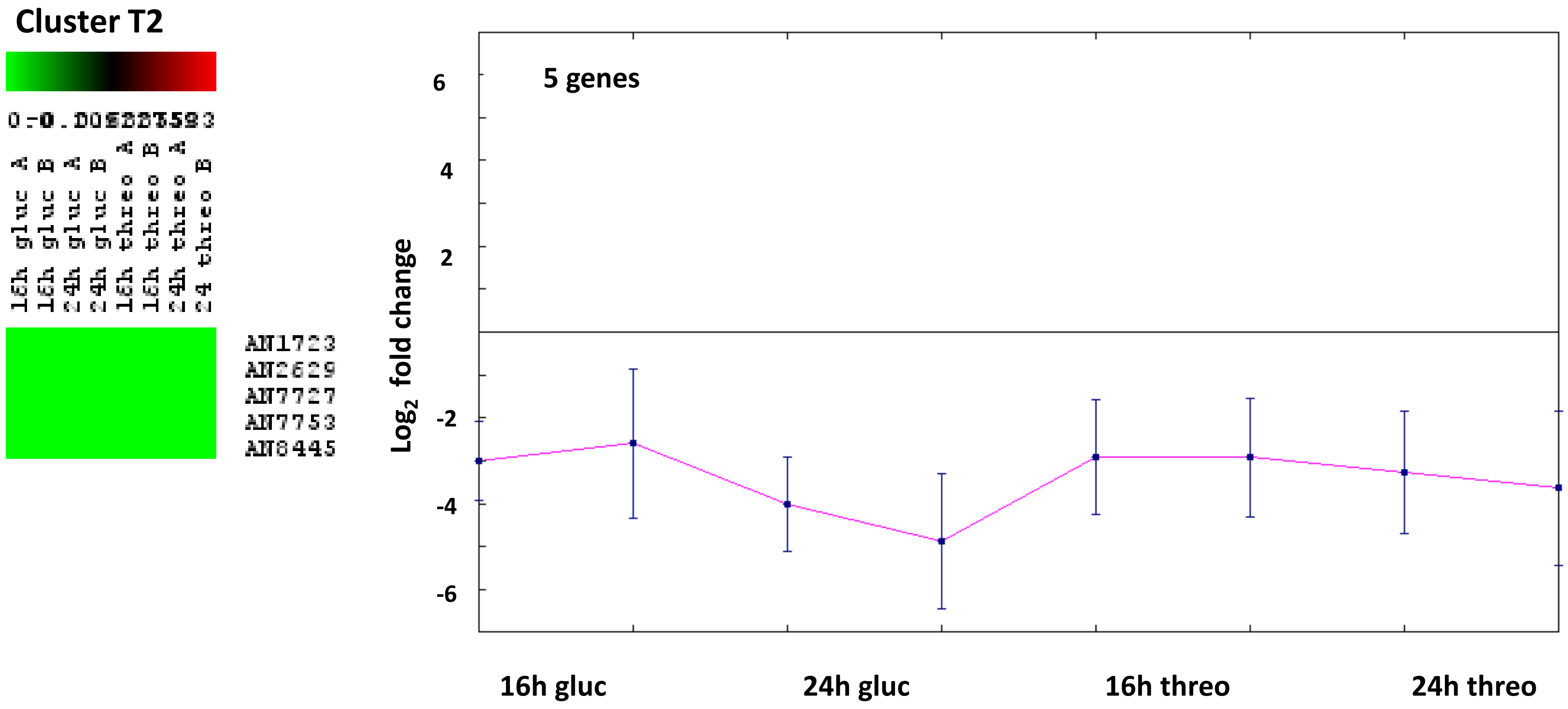

Cluster T2
16h gluc A
16h gluc B
24h gluc A
24h gluc B
16h threo A
16h threo B
24h threo A
24 threo B
AN1723
AN2629
AN7727
AN7753
AN8445
5 genes
6
4
2
-2
-4
-6
Log2 fold change
16h gluc
24h gluc
16h threo
24h threo

Cluster T3

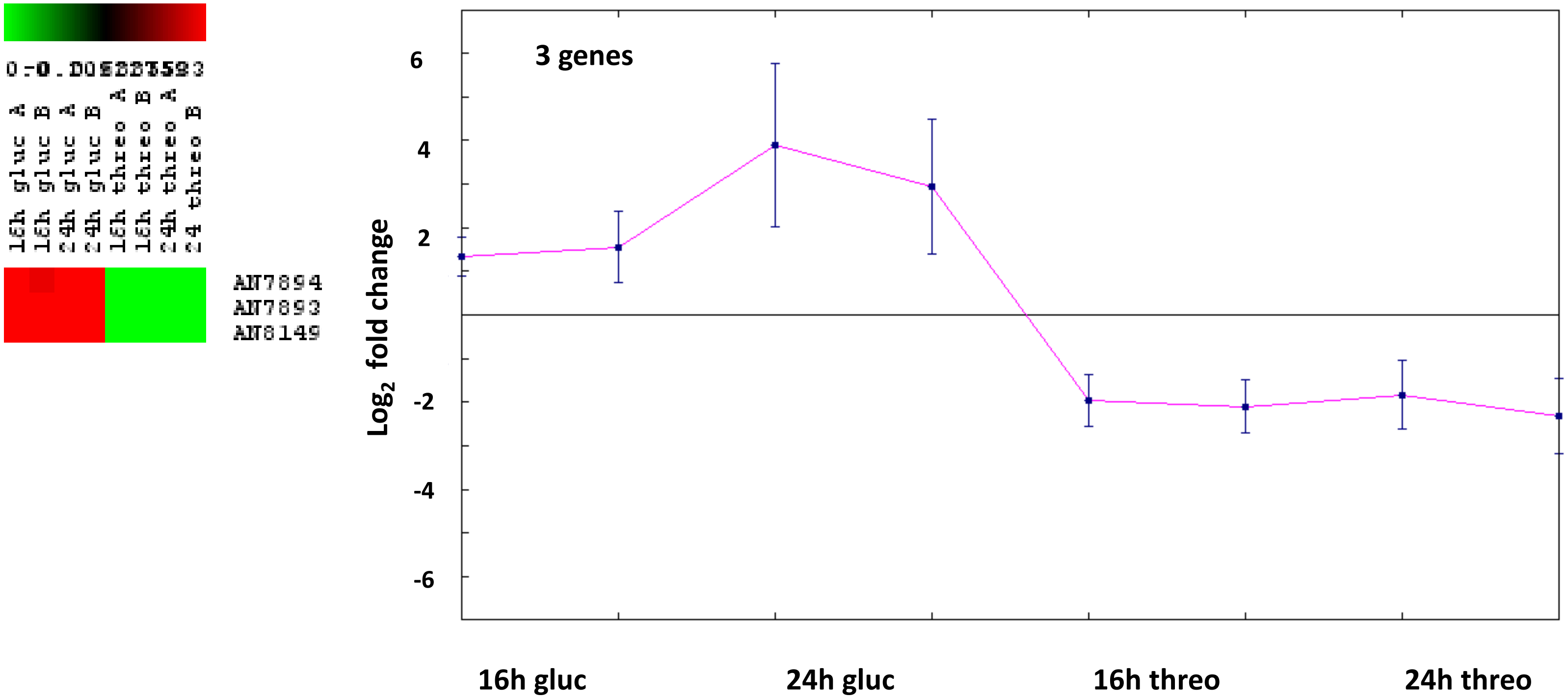
16h gluc A
16h gluc B
24h gluc A
24h gluc B
16h threo A
16h threo B
24h threo A
24 threo B
AN7894
AN7893
AN8149
3 genes
Log2 fold change
6
4
2
-2
-4
-6
16h gluc
24h gluc
16h threo
24h threo

Cluster T4

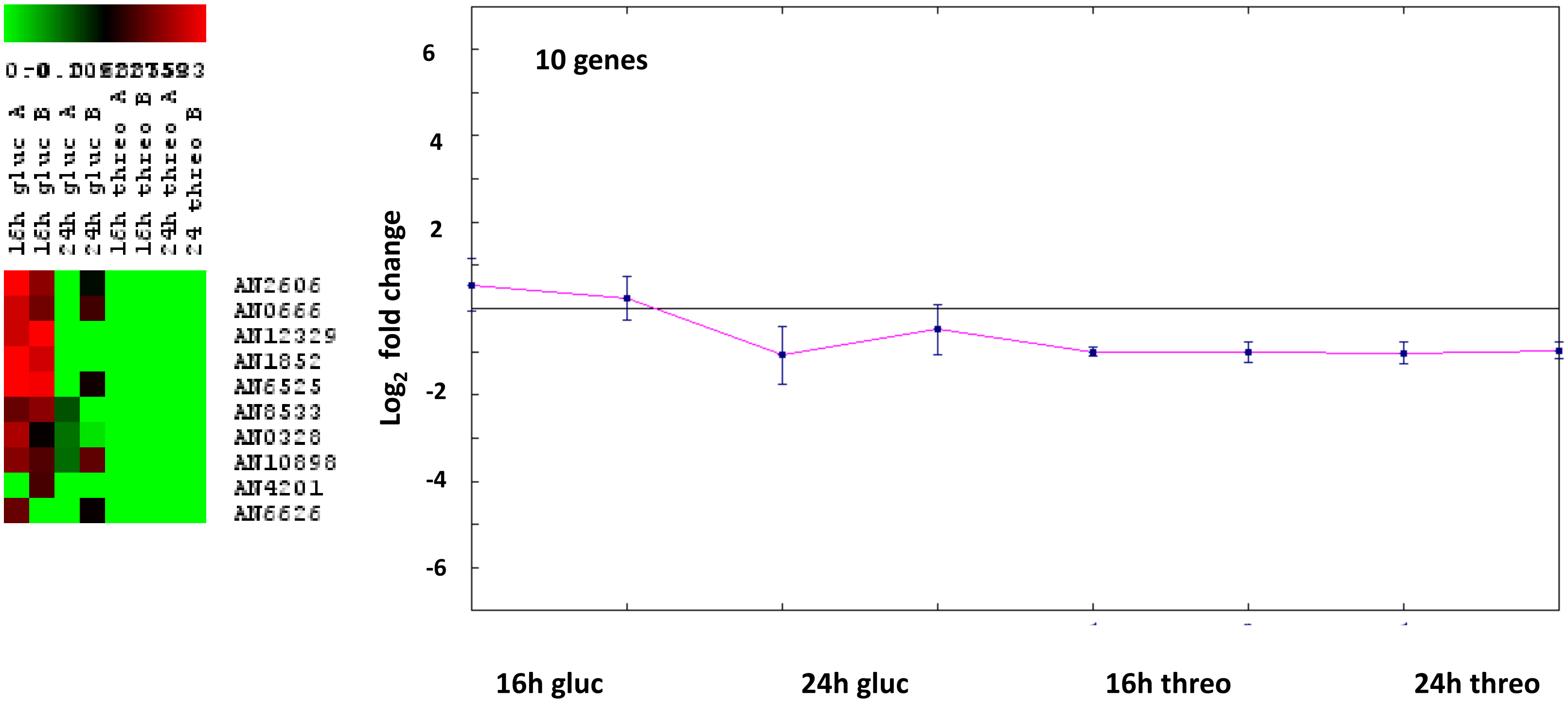
16h gluc A
16h gluc B
24h gluc A
24h gluc B
16h threo A
16h threo B
24h threo A
24 threo B
AN2606
AN0666
AN12329
AN1852
AN6525
AN8533
AN0328
AN10898
AN4201
AN6626
10 genes
Log2 fold change
6
4
2
-2
-4
-6
16h gluc
24h gluc
16h threo
24h threo

Cluster T5

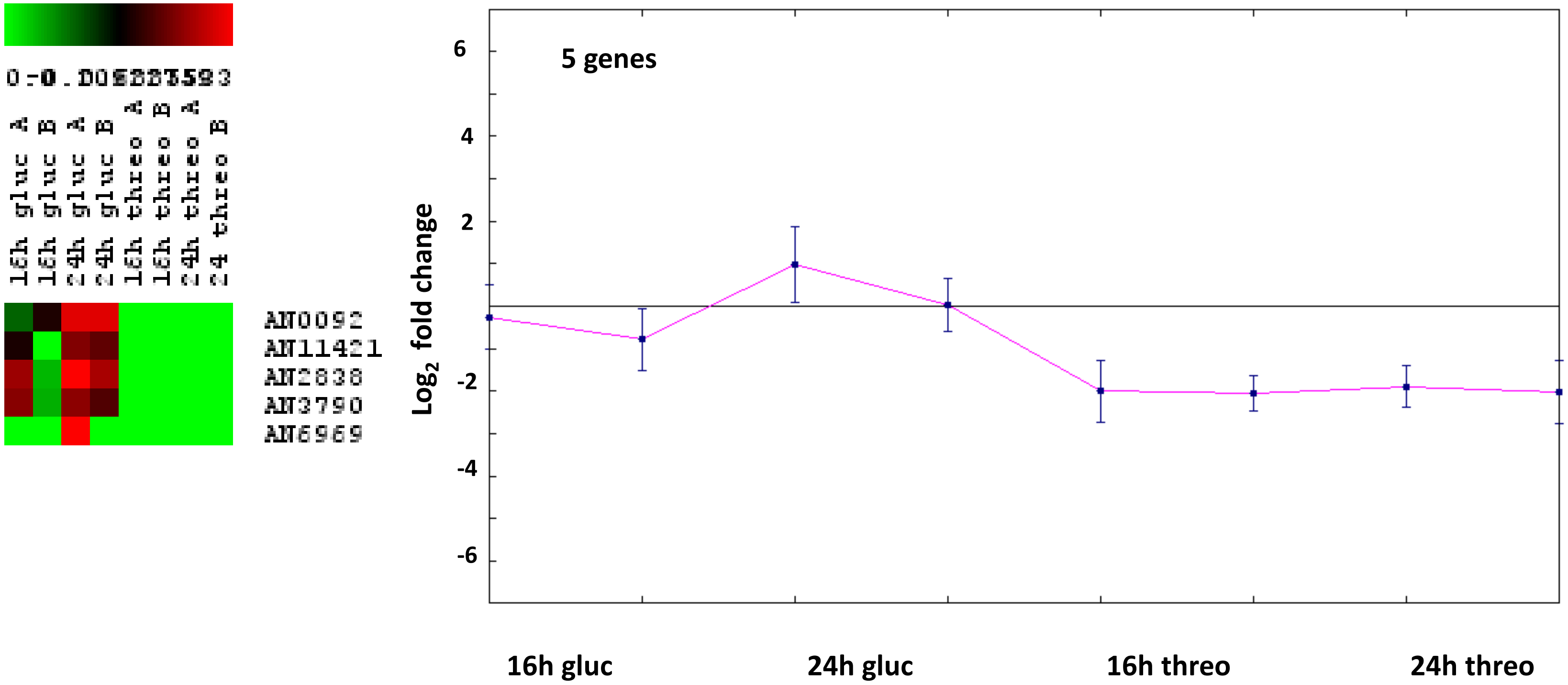
16h gluc A
16h gluc B
24h gluc A
24h gluc B
16h threo A
16h threo B
24h threo A
24 threo B
AN0092
AN11421
AN2838
AN3790
AN6969
5 genes
6
4
2
-2
-4
-6
Log2 fold change
16h gluc
24h gluc
16h threo
24h threo

Cluster T6

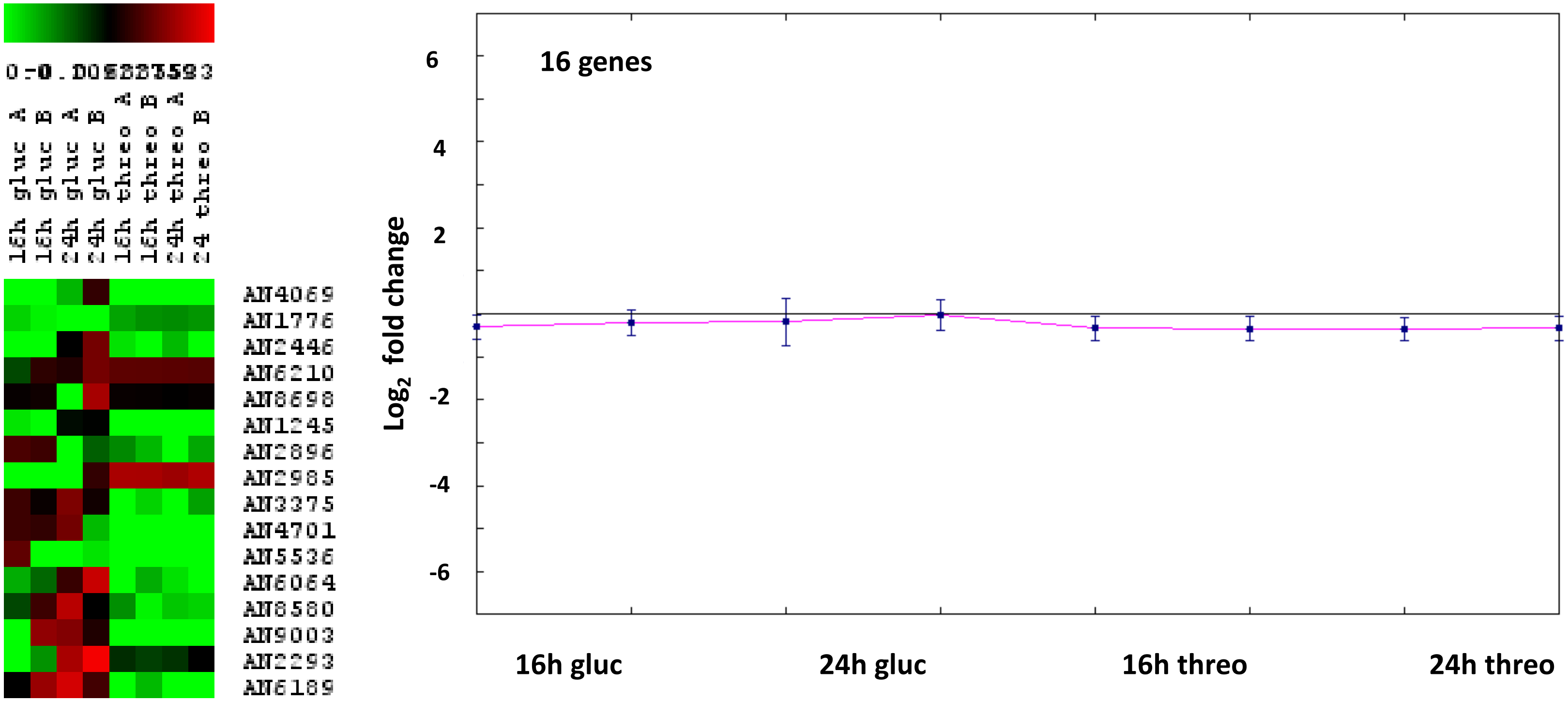
16h gluc A
16h gluc B
24h gluc A
24h gluc B
16h threo A
16h threo B
24h threo A
24 threo B
AN4069
AN1776
AN2446
AN6210
AN8698
AN1245
AN2896
AN2985
AN3375
AN4701
AN5536
AN6064
AN8580
AN9003
AN2293
AN6189
16 genes
Log_2 fold change
6
4
2
-2
-4
-6
16h gluc
24h gluc
16h threo
24h threo

Cluster T7

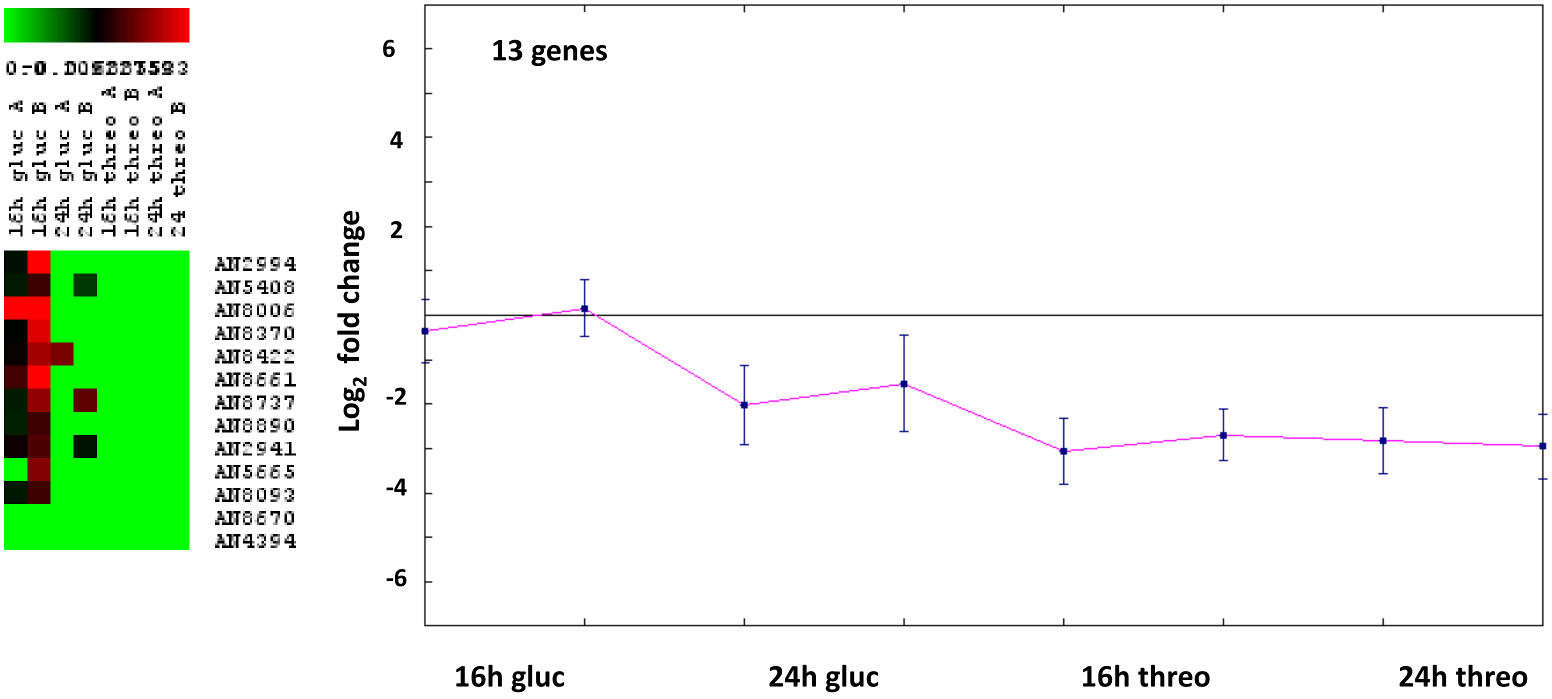
16h gluc A
16h gluc B
24h gluc A
24h gluc B
16h threo A
16h threo B
24h threo A
24 threo B
AN2994
AN5408
AN8006
AN8370
AN8422
AN8661
AN8737
AN8890
AN2941
AN5665
AN8093
AN8670
AN4394
13 genes
6
4
2
-2
-4
-6
Log2 fold change
16h gluc
24h gluc
16h threo
24h threo

Cluster T8

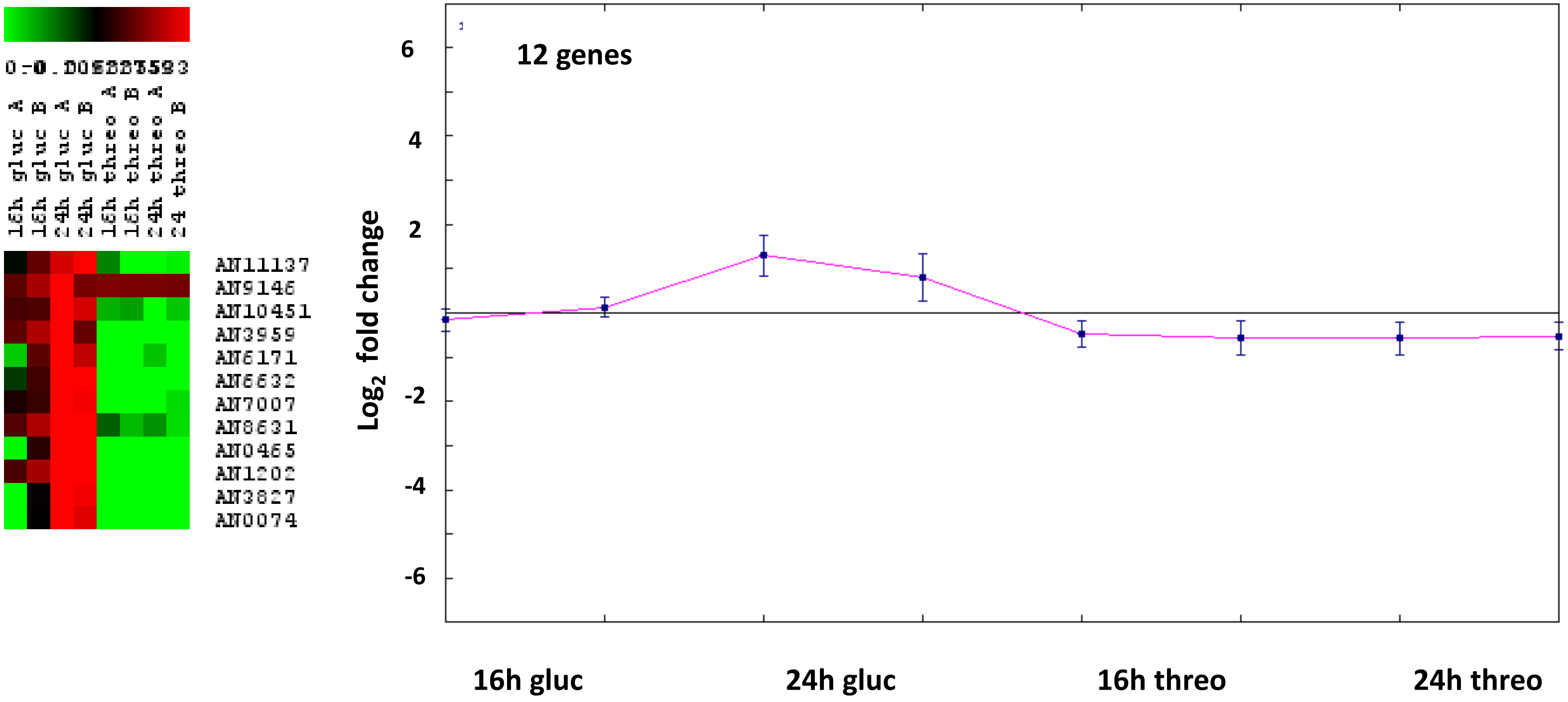
16h gluc A
16h gluc B
24h gluc A
24h gluc B
16h threo A
16h threo B
24h threo A
24 threo B
AN11137
AN9146
AN10451
AN3959
AN6171
AN6632
AN7007
AN8631
AN0465
AN1202
AN3827
AN0074
12 genes
6
4
2
-2
-4
-6
Log_2 fold change
16h gluc
24h gluc
16h threo
24h threo

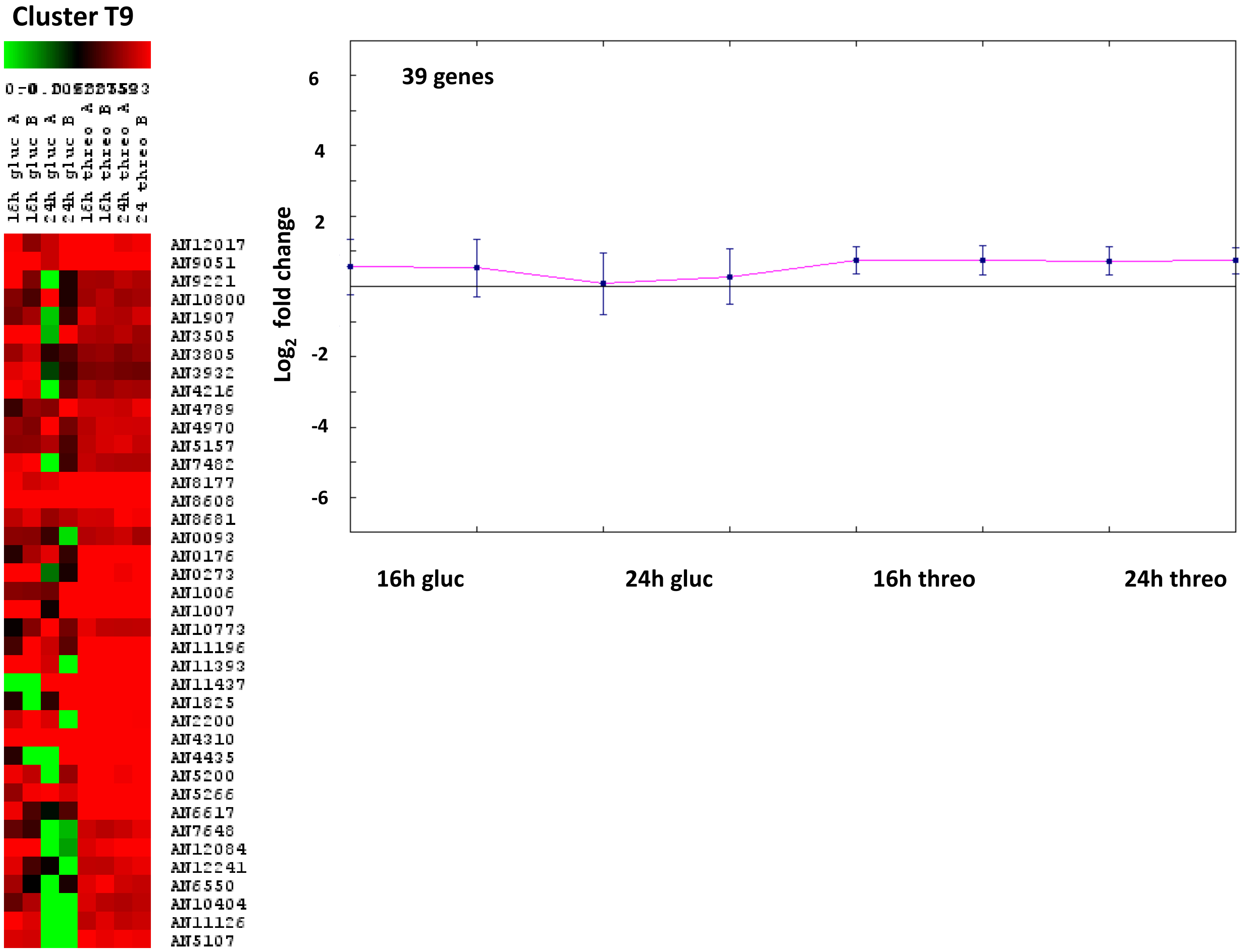

Cluster T9
16h gluc A
16h gluc B
24h gluc A
24h gluc B
16h threo A
16h threo B
24h threo A
24 threo B
AN12017
AN9051
AN9221
AN10800
AN1907
AN3505
AN3805
AN3932
AN4216
AN4789
AN4970
AN5157
AN7482
AN8177
AN8608
AN8681
AN0093
AN0176
AN0273
AN1006
AN1007
AN10773
AN11196
AN11393
AN11437
AN1825
AN2200
AN4310
AN4435
AN5200
AN5266
AN6617
AN7648
AN12084
AN12241
AN6550
AN10404
AN11126
AN5107
39 genes
Log2 fold change
6
4
2
-2
-4
-6
16h gluc
24h gluc
16h threo
24h threo

Cluster T10

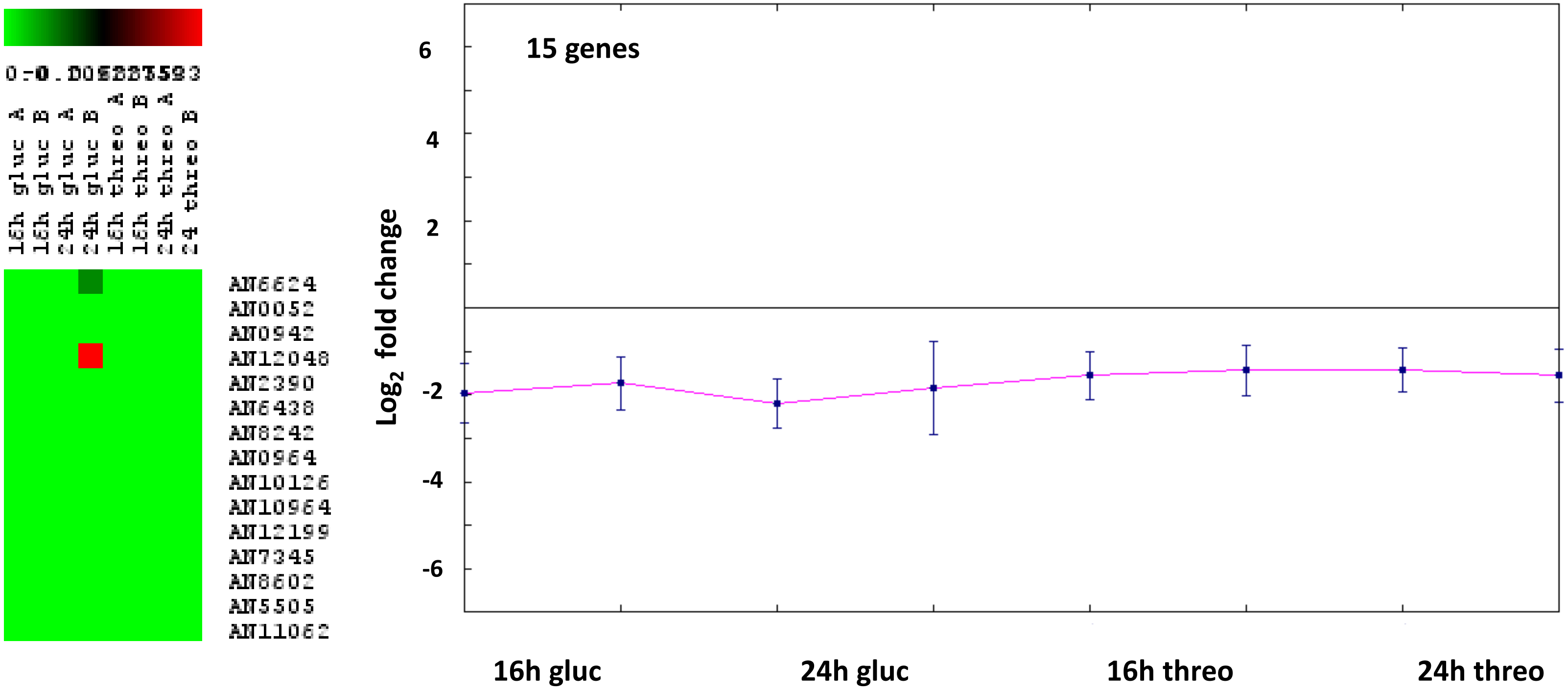
16h gluc A
16h gluc B
24h gluc A
24h gluc B
16h threo A
16h threo B
24h threo A
24 threo B
AN6624
AN0052
AN0942
AN12048
AN2390
AN6438
AN8242
AN0964
AN10126
AN10964
AN12199
AN7345
AN8602
AN5505
AN11062
15 genes
Log2 fold change
6
4
2
-2
-4
-6
16h gluc
24h gluc
16h threo
24h threo
