# Supplementary material for: Functional Characterization of Aspergillus nidulans ypkA, a Homologue of the Mammalian Kinase SGK
Source: PLoS One. 2013 Mar 5;8(3):e57630. doi: 10.1371/journal.pone.0057630 (PMC3589345; doi:10.1371/journal.pone.0057630)
Supplement: Table S3 — Strains used in this work. (DOCX) [file pone.0057630.s004.pdf]

Table S3. Strains used in this work.

| **Strain** | **Genotype** | **Reference** |
| --- | --- | --- |
| *alca::ypkA* | *pyroA4; chaA1; alcA::ypkA* | This work |
| *niiA::ypkA* | *pyroA4; chaA1; argB2; Δnku::argB; niiA::ypkA::pyrG* | This work |
| *niiA::pkh1* | *pyroA4; chaA1; argB2; Δnku::argB; niiA::pkh1::pyrG* | This work |
| *barA1* | \| pabaA6; biA1; barA1, veA1 \| \| --- \| | Li et al., 2006 |
| *niiA::ypkA barA1* | *barA1 niiA::ypkA::pyrG* | This work |
| *niiA::pkh1 alcA::ypkA* | *niiA::pkh1 alcA::ypkA* | This work |
| *alcA::tubC::mRFP* | *pyroA4; chaA1; argB2; Δnku::argB; alcA::tubC::mRFP pyyG* | This work |
| *GFP::ypkA* | *pyroA4; chaA1; argB2; Δnku::argB; GFP::ypkA::pyrG* | This work |
| *GFP::ypkA alcA::tubC::mRFP* | *GFP::ypkA alcA::tubC::mRFP* | This work |
| *alcA::pkcA* | *pyroA4; chaA1; alcA::pkcA* | This work |
| GR5 | *pyroA4 pyrG89, wA3* | FGSCA773 |
| TNO2A3 | *pyroA4 pyrG89; chaA1;* Δ*nKuA::argB* | NAYAK et al., 2006 |
| *PilA* GFP | pyrG89 argB2 nkuAΔ::argB^+^ pabaB22 pilA::sgfp::AfpyrG^+^ riboB2 | Vangelatos I, et al 2010 |
| *PilB* GFP | pyrG89 pilB::sgfp::AfpyrG^+^ argB2 nkuAΔ::argB^+^ pabaB22 riboB2 | Vangelatos I, et al 2010 |
