# Supplementary material for: Functional Characterization of Aspergillus nidulans ypkA, a Homologue of the Mammalian Kinase SGK
Source: PLoS One. 2013 Mar 5;8(3):e57630. doi: 10.1371/journal.pone.0057630 (PMC3589345; doi:10.1371/journal.pone.0057630)
Supplement: Table S4 — Primers and probes used in this work. (DOCX) [file pone.0057630.s005.pdf]

Table S4. Primers and probes used in this work.

| **Name** | **Sequence 5’🡪 3’** |  |
| --- | --- | --- |
| *ypkA*-1 | GGCAGCTTCGGAAAGGTCAT |  |
| *ypkA*-2 | CGGGGAGAGCATAGATACGGCC[FAM]G |  |
| *tubC*-1 | GCAGAATGTCTCGTCCGAATG |  |
| *tubC*-2 | CACTTTATGCCGTCGCCGAAAG[FAM]G |  |
| AN6669-1 | GGTCTCCTCCATCATCTTGTCG |  |
| AN6669-2 | CGGGCTACACCTACTTCCTGATTCC[FAM]G |  |
| AN8737-1 | CAGAGGAGTTGGAGGGCAATC |  |
| AN8737-2 | CGGTTGAACTATGGGACGGAGAAC[FAM]G |  |
| 5’pkcB pRS426 FWD | GGTTTTCCCAGTCACGACGGTTTTCTCTCCGGCCGCGAAG | |
| 5’ pkcB pyr4 RV | TCATCACCGAAACGCGCGATGTGAAGGCGGCGGTCTGG | |
| pkcB pRS426 RV | CAATTTCACACAGGAAACAGCGGCGAATGATGGATCCTTGACAC | |
| Del pkcB chec | GCTCCCACCAGCTTTCATTGATTCC | |
| Del pkcB 1 | TTCCCAGTCACGACGACCTTGCACCTCATGACAAGC | |
| Del pkcB 1 RV | AACAACCATGATACCATGTGAAGGCGGCGGTCTGG | |
| Del pkcB 2 | CGATCATGTGGATGCTACATCCGCGACGGTCTGGCT | |
| Del pkcB 2 RV | CACACAGGAAACAGCGGCGGACTTGCGAAGAGC | |
| An pkcB FW | GGCGCGCCAATGTCCTGGAAGCTTACTAAAAAGC | |
| An pkcB RV | CCTTAATTAAGCTTTACGAATGGTCTTGAGAGC | |
| pyrG K7 niiA FW | GCAGGGAAAAATACGAGCTCC | |
| niiA K7 niiA | CGTGACGAAGTCTCAACGCC | |
| Spacer GFP | GGAACACGGGGAATGAGTAAAGGAGAAGAACT | |
| GFPVE3’AF | CTCAGACAGAATACGCCAAGCTTGCATGC | |
| 5 pkcB niiA RV | CGTATTTTTCCCTGCTGTGAAGGCGGCGGTCTG | |
| ORF pkcB niiA FW | CGTTGAGACTTCGTCACGATGTCCTGGAAGCTTACTAAAAGTATG | |
| ORF pkcB pRS426 | CAATTTCACACAGGAAACAGCAGGCTACGGTGCTTGCTCTCAAC | |
| pkcB spacer GFP Rev | CTTTACTCATTCCCCGTGTTCCGAATCGAGAATCCTCAGG | |
| Pyr 3’ pkcB Rev | TCGCGGATGTGAATTCGCCTCAAACAATGC | |
| 3’ pkcB pyr Fwd | TGAGGCGAATTCACATCCGCGACGGTCTGGCT | |
| niiApkh1 5 RV | CGTATTTTTCCCCTGCACTAGACGCGACAATCC | |
| niiApkh1 5 FW | CAGCGTTTGCGACTGAACC | |
| niiApkh1 orf FW | GAGACTTCGTCACGATGGATGGGGATATTAG | |
| niiApkh1 orf RV | CCGCTCGAATTCTGAGAGGCTT | |
